# Supplementary material for: Cerebrospinal fluid metabolomics identifies 19 brain-related phenotype associations
Source: Commun Biol. 2021 Jan 12;4:63. doi: 10.1038/s42003-020-01583-z (PMC7803963; doi:10.1038/s42003-020-01583-z)
Supplement: Supplementary file 2 — Supplementary Figures [file 42003_2020_1583_MOESM2_ESM.pdf]

Table of Contents

Supplementary Figure 1. Overview of MWAS 2
Supplementary Figure 2. GWAS meta-analysis results for methionine sulfone (X44748) 3
Supplementary Figure 3. GWAS meta-analysis results for N-delta-acetylornithine (X43249) 4
Supplementary Figure 4. GWAS meta-analysis results for bilirubin (X43807) 5
Supplementary Figure 5. GWAS meta-analysis results for betaine (X3141) 6
Supplementary Figure 6. GWAS meta-analysis results for oxalate (ethanedioate) (X20694) 7
Supplementary Figure 7. GWAS meta-analysis results for tryptophan betaine (X37097) 8
Supplementary Figure 8. GWAS meta-analysis results for 2-hydroxyadipate (X31934) 9
Supplementary Figure 9. GWAS meta-analysis results for N-acetylhistidine (X33946) 10
Supplementary Figure 10. GWAS meta-analysis results for 3-ureidopropionate (X3155) 11
Supplementary Figure 11. GWAS meta-analysis results for 1-ribosyl-imidazoleacetate (X61868) 12
Supplementary Figure 12. GWAS meta-analysis results for 2'-O-methylcytidine (X57554) 13
Supplementary Figure 13. GWAS meta-analysis results for N-acetyl-beta-alanine (X37432) 14
Supplementary Figure 14. GWAS meta-analysis results for N6-methyllysine (X62249) 15
Supplementary Figure 15. GWAS meta-analysis results for ethylmalonate (X15765) 16
Supplementary Figure 16. GWAS meta-analysis results for guanosine (X1573) 17
Supplementary Figure 17. GWAS meta-analysis results for N-acetylglutamate (X15720) 18
Supplementary Figure 18. Genomic inflation factor distribution 19
Supplementary Figure 19. Side-by-side regional association plots for methionine sulfone (X44878) 20
Supplementary Figure 20. Side-by-side regional association plots for N-delta-acetylornithine (X43249) 21
Supplementary Figure 21. Side-by-side regional association plots for bilirubin (X43807) 22
Supplementary Figure 22. Side-by-side regional association plots for tryptophan betaine (X37097) 23
Supplementary Figure 23. Side-by-side regional association plots for N-acetylhistidine (X33946) 24
Supplementary Figure 24. Side-by-side regional association plots for N-acetyl-beta-alanine (X37432) ... 25
Supplementary Figure 25. Side-by-side regional association plots for ethylmalonate (X15765) 26
Supplementary Figure 26. Metabolite prediction model building process 27
Supplementary Figure 27. Metabolite prediction model performance by model type 28
Supplementary Figure 28. Metabolite-phenotype association analysis Q-Q plot 29
Supplementary Figure 29. Two-sample Mendelian Randomization model set-up 30
Supplementary References 31

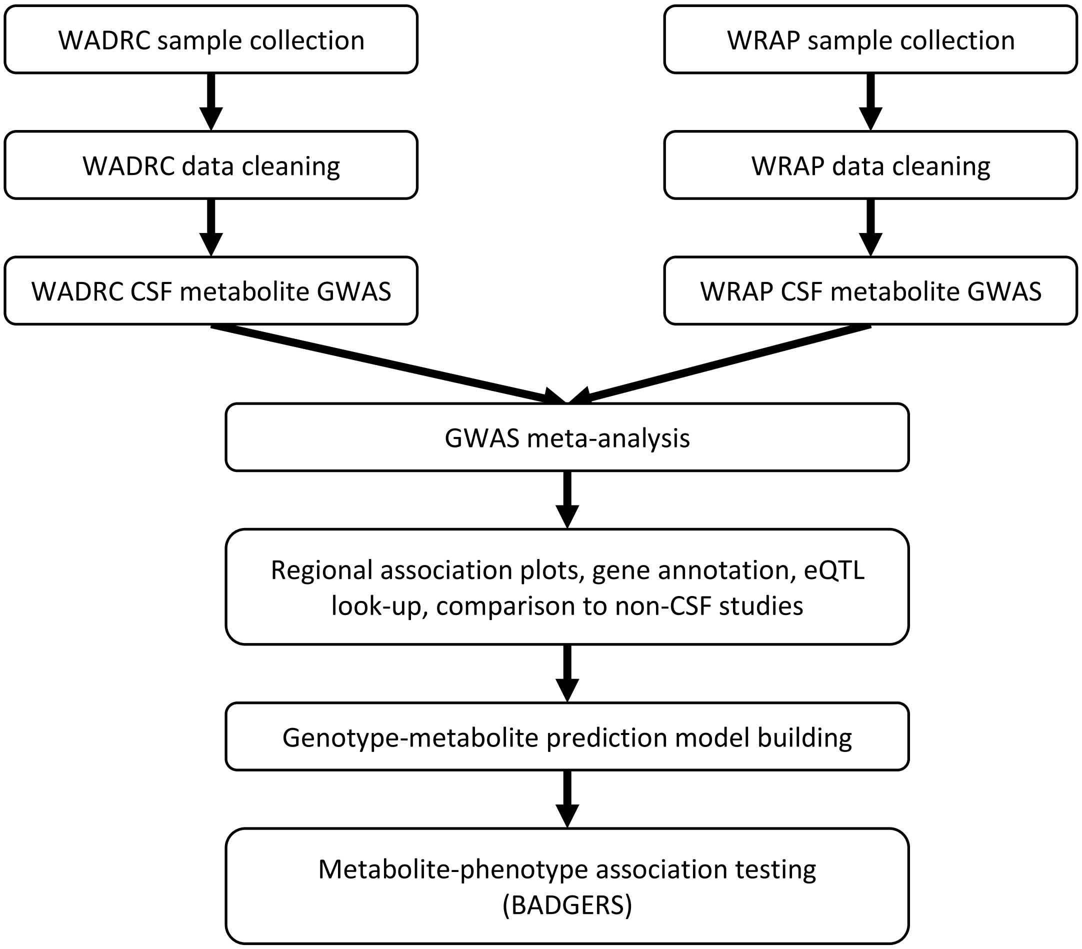

Supplementary Figure 1. Overview of MWAS

The flow chart highlights the main sequence of steps in the MWAS methodology as applied in this paper. After strict genotype and metabolite quality control, a GWAS of each CSF metabolite was performed, first in WADRC as a discovery cohort and then in WRAP as a replication cohort. The results were meta-analyzed together and the resulting SNP-metabolite loci reviewed for feasibility with regional association plots, gene and eQTL annotation, and comparison to previous non-CSF metabolomics GWAS. The combined WADRC/WRAP data set was then used to build metabolite prediction models from genotypes, and the resulting models were used to test for metabolite-phenotype associations with neurological and psychiatric phenotypes in a summary-statistic-based TWAS-like method (BADGERS).

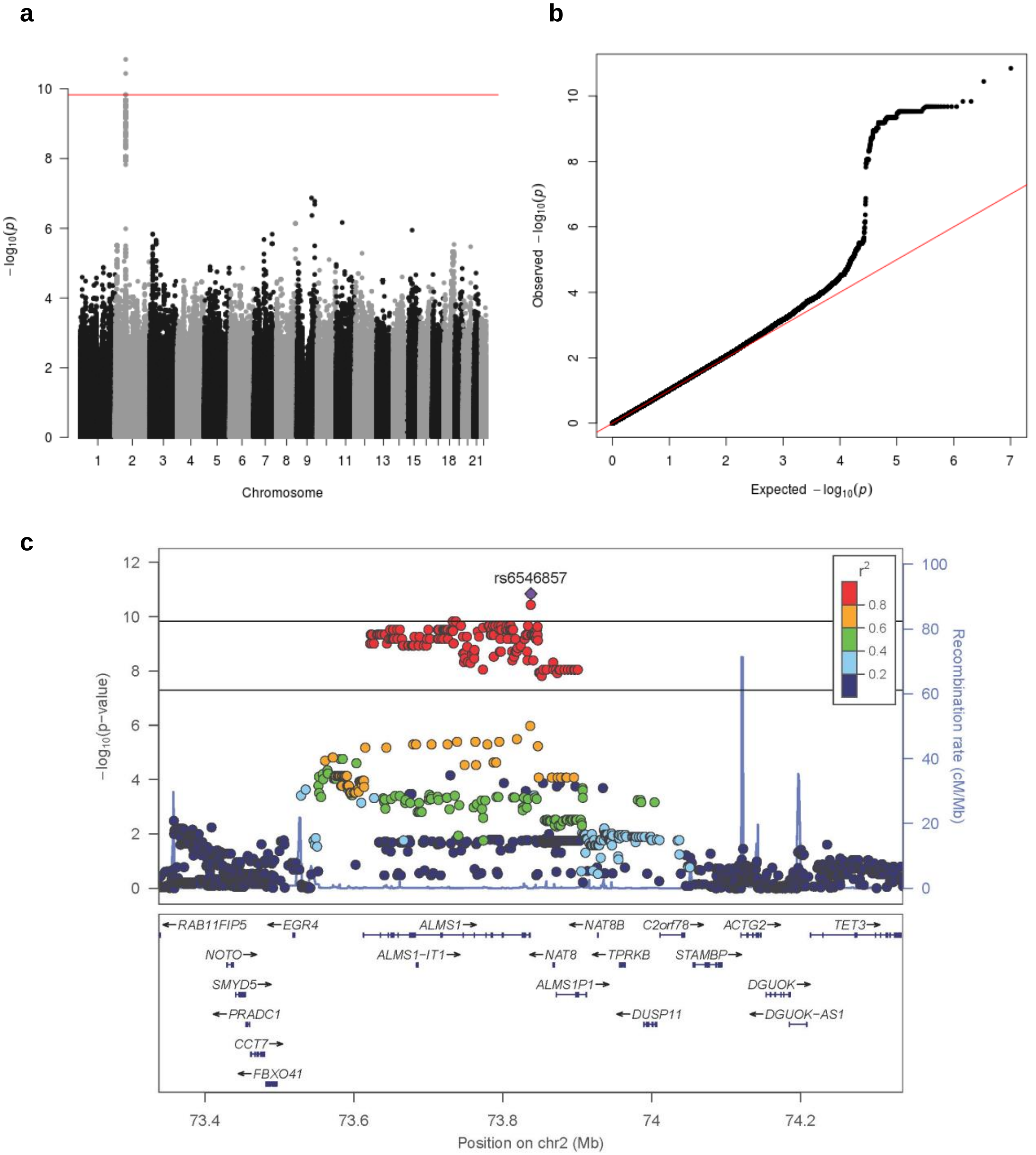

Supplementary Figure 2. GWAS meta-analysis results for methionine sulfone (X44748)

a) The Manhattan plot, with a red line indicating the Bonferroni-corrected significance threshold (P = 1.48×10^{-10}). **b)** The Q-Q plot of the estimated effect sizes. **c)** The regional association plot for the associated locus. The top horizontal line represents the Bonferroni-corrected p-value threshold while the bottom line represents the standard genome-wide significance threshold.

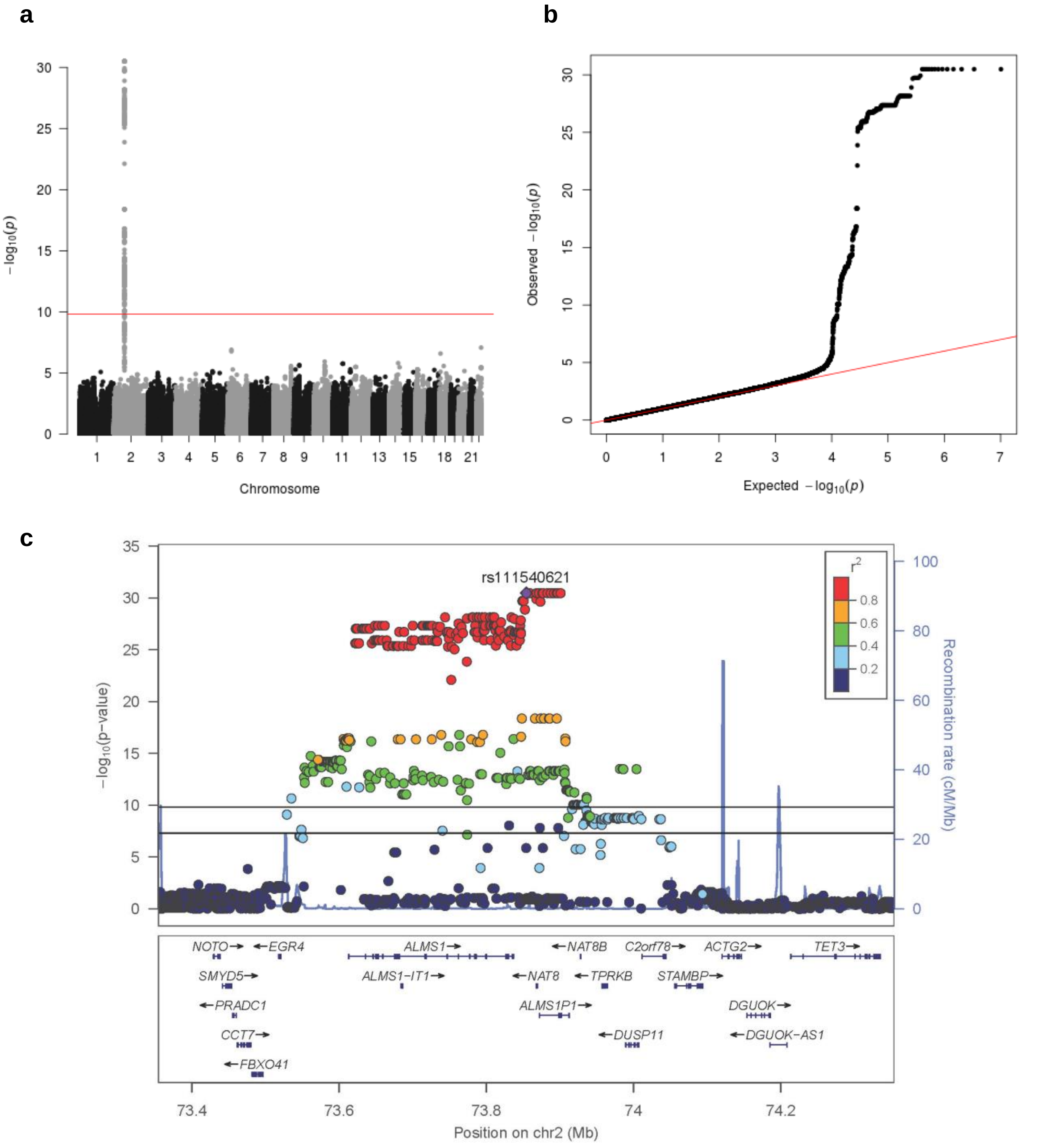

Supplementary Figure 3. GWAS meta-analysis results for N-delta-acetylornithine (X43249)

a) The Manhattan plot, with a red line indicating the Bonferroni-corrected significance threshold (P = 1.48×10^{-10}). **b)** The Q-Q plot of the estimated effect sizes. **c)** The regional association plot for the associated locus. The top horizontal line represents the Bonferroni-corrected p-value threshold while the bottom line represents the standard genome-wide significance threshold.

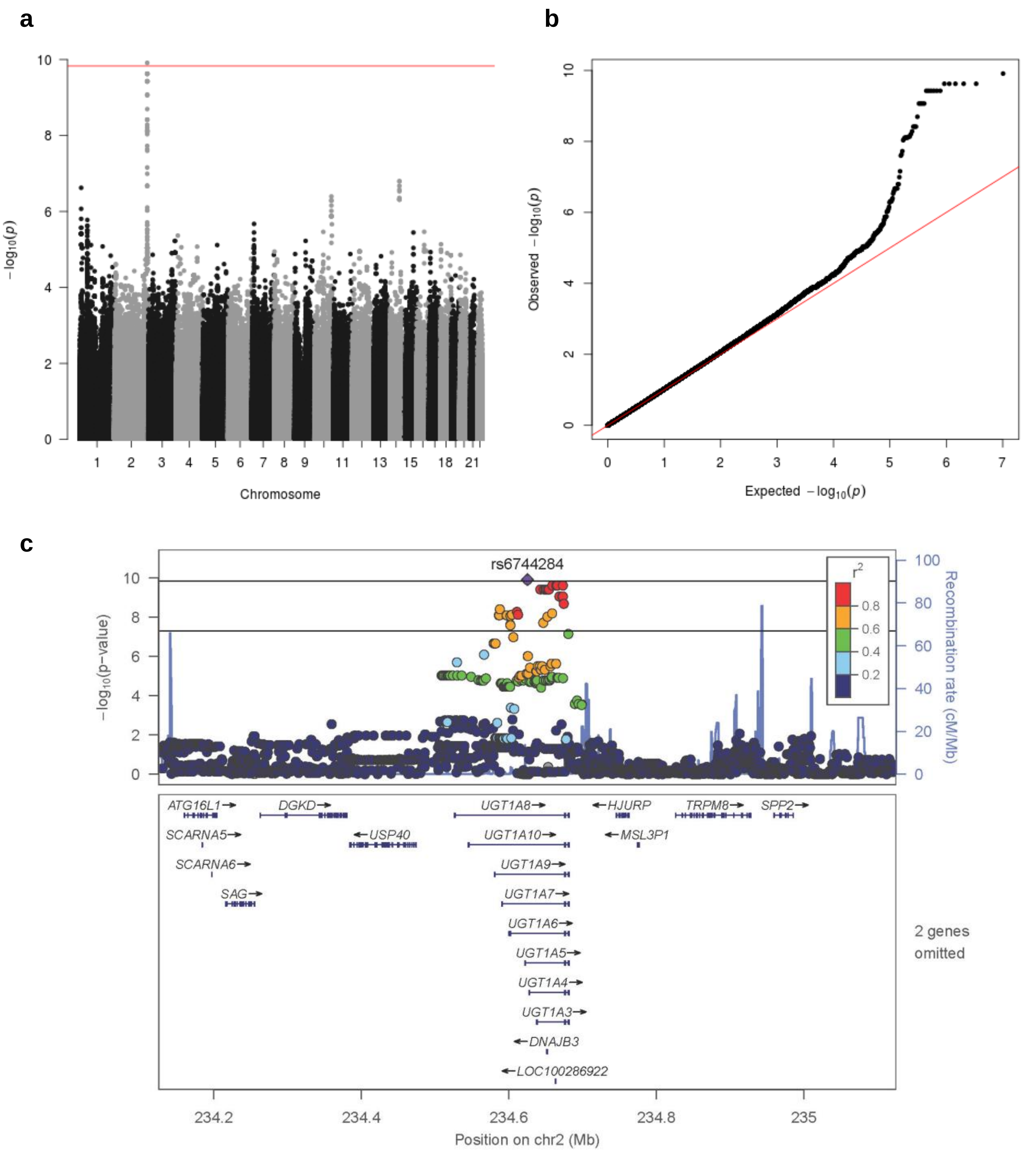

Supplementary Figure 4. GWAS meta-analysis results for bilirubin (X43807)

a) The Manhattan plot, with a red line indicating the Bonferroni-corrected significance threshold (P = 1.48×10^{-10}). **b)** The Q-Q plot of the estimated effect sizes. **c)** The regional association plot for the associated locus. The top horizontal line represents the Bonferroni-corrected p-value threshold while the bottom line represents the standard genome-wide significance threshold.

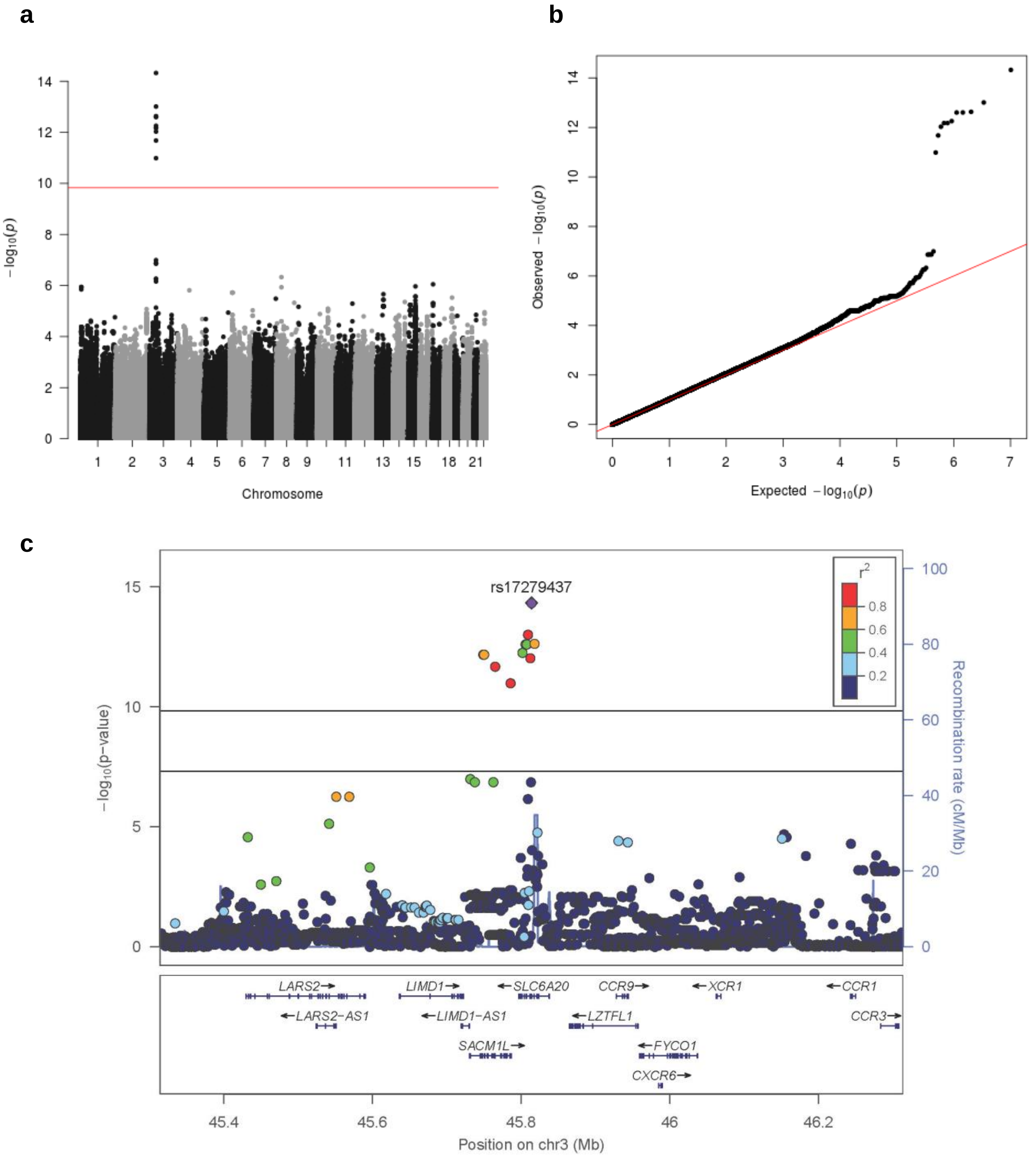

Supplementary Figure 5. GWAS meta-analysis results for betaine (X3141)

a) The Manhattan plot, with a red line indicating the Bonferroni-corrected significance threshold (P = 1.48×10^{-10}). **b)** The Q-Q plot of the estimated effect sizes. **c)** The regional association plot for the associated locus. The top horizontal line represents the Bonferroni-corrected p-value threshold while the bottom line represents the standard genome-wide significance threshold.

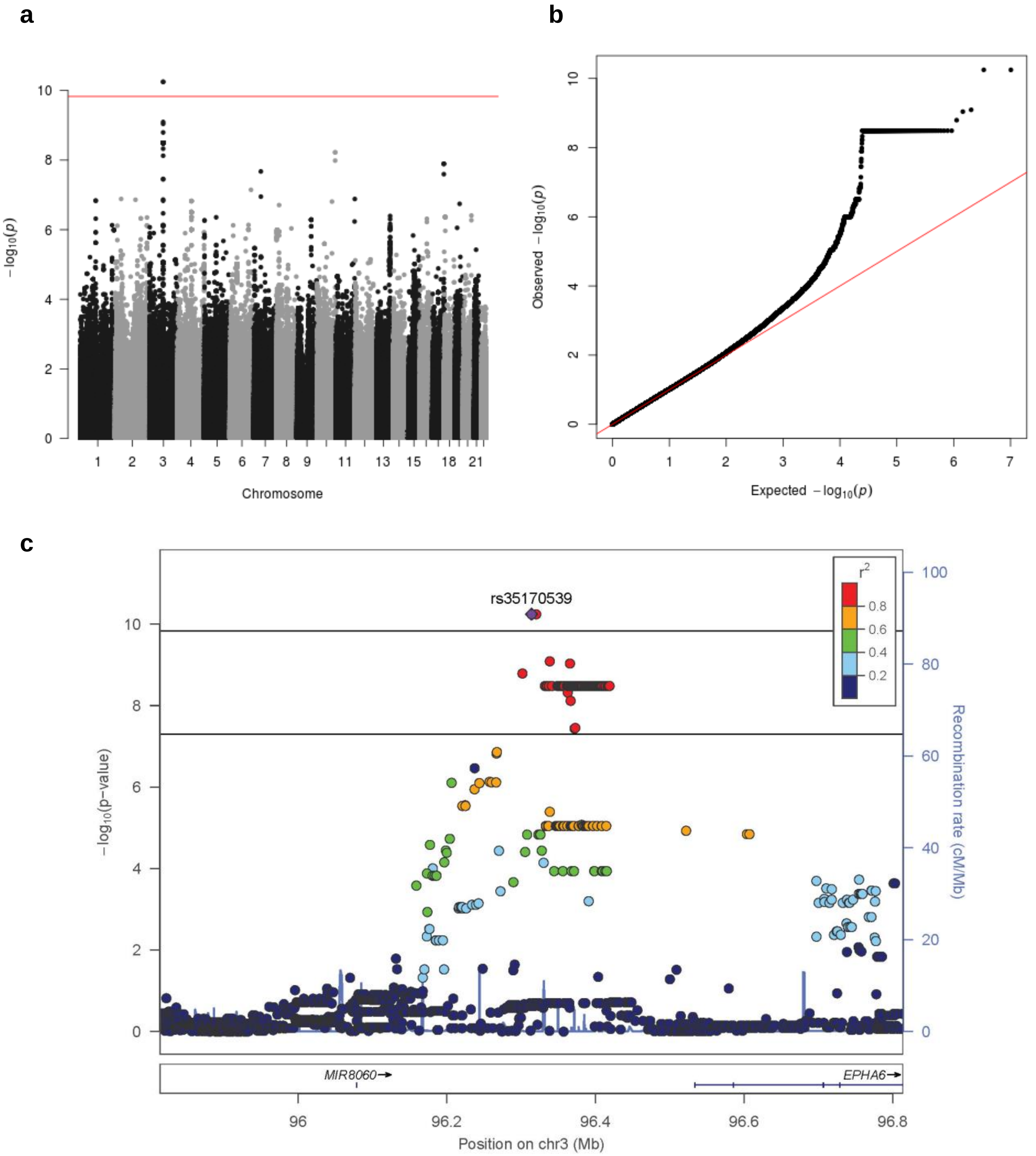

Supplementary Figure 6. GWAS meta-analysis results for oxalate (ethanedioate) (X20694)

a) The Manhattan plot, with a red line indicating the Bonferroni-corrected significance threshold (P = 1.48×10^{-10}). **b)** The Q-Q plot of the estimated effect sizes. **c)** The regional association plot for the associated locus. The top horizontal line represents the Bonferroni-corrected p-value threshold while the bottom line represents the standard genome-wide significance threshold.

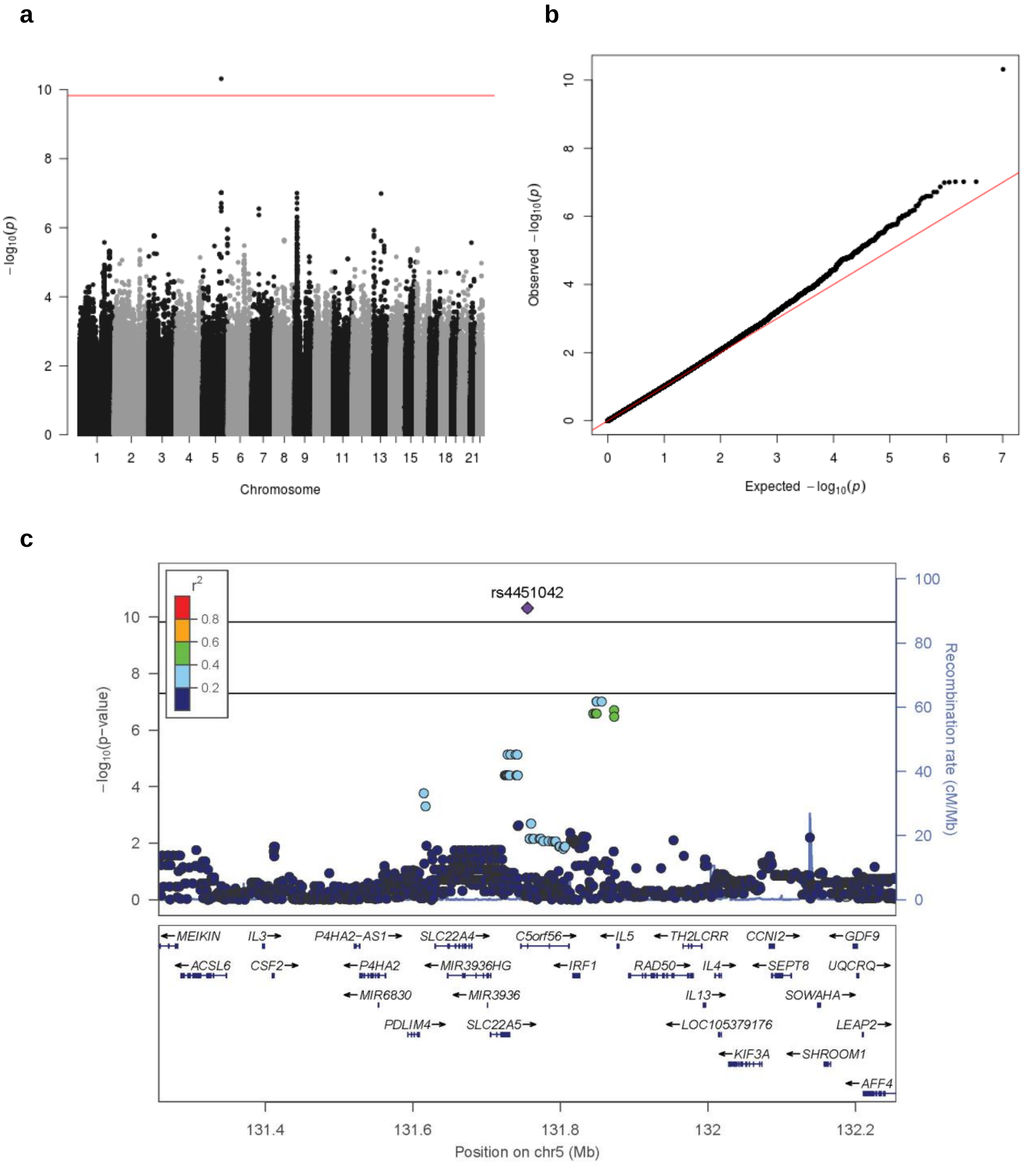

Supplementary Figure 7. GWAS meta-analysis results for tryptophan betaine (X37097)

a) The Manhattan plot, with a red line indicating the Bonferroni-corrected significance threshold (P = 1.48×10^{-10}). **b)** The Q-Q plot of the estimated effect sizes. **c)** The regional association plot for the associated locus. The top horizontal line represents the Bonferroni-corrected p-value threshold while the bottom line represents the standard genome-wide significance threshold.

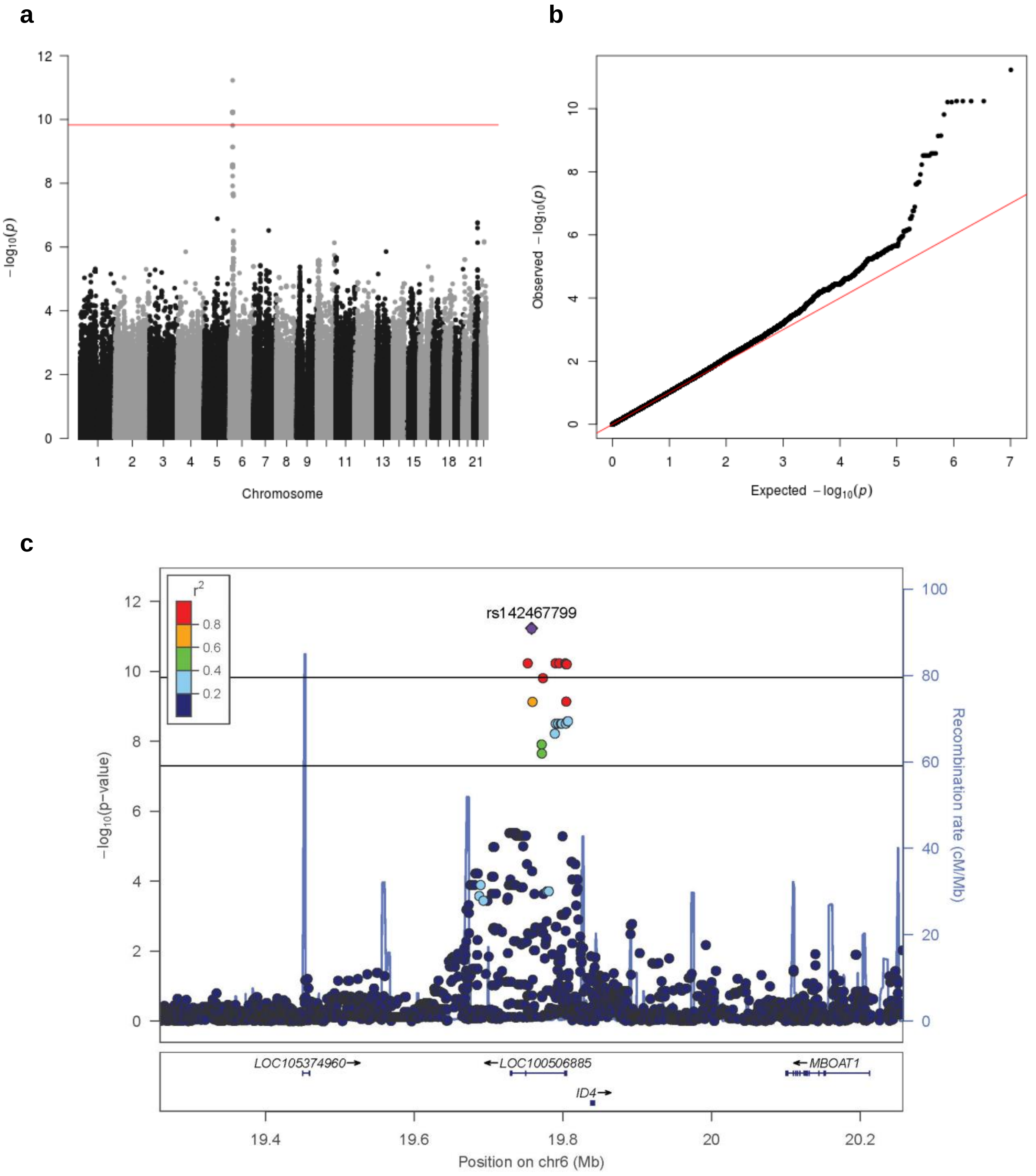

Supplementary Figure 8. GWAS meta-analysis results for 2-hydroxyadipate (X31934)

a) The Manhattan plot, with a red line indicating the Bonferroni-corrected significance threshold (P = $1.48x10^{-10}$). **b)** The Q-Q plot of the estimated effect sizes. **c)** The regional association plot for the associated locus. The top horizontal line represents the Bonferroni-corrected p-value threshold while the bottom line represents the standard genome-wide significance threshold.

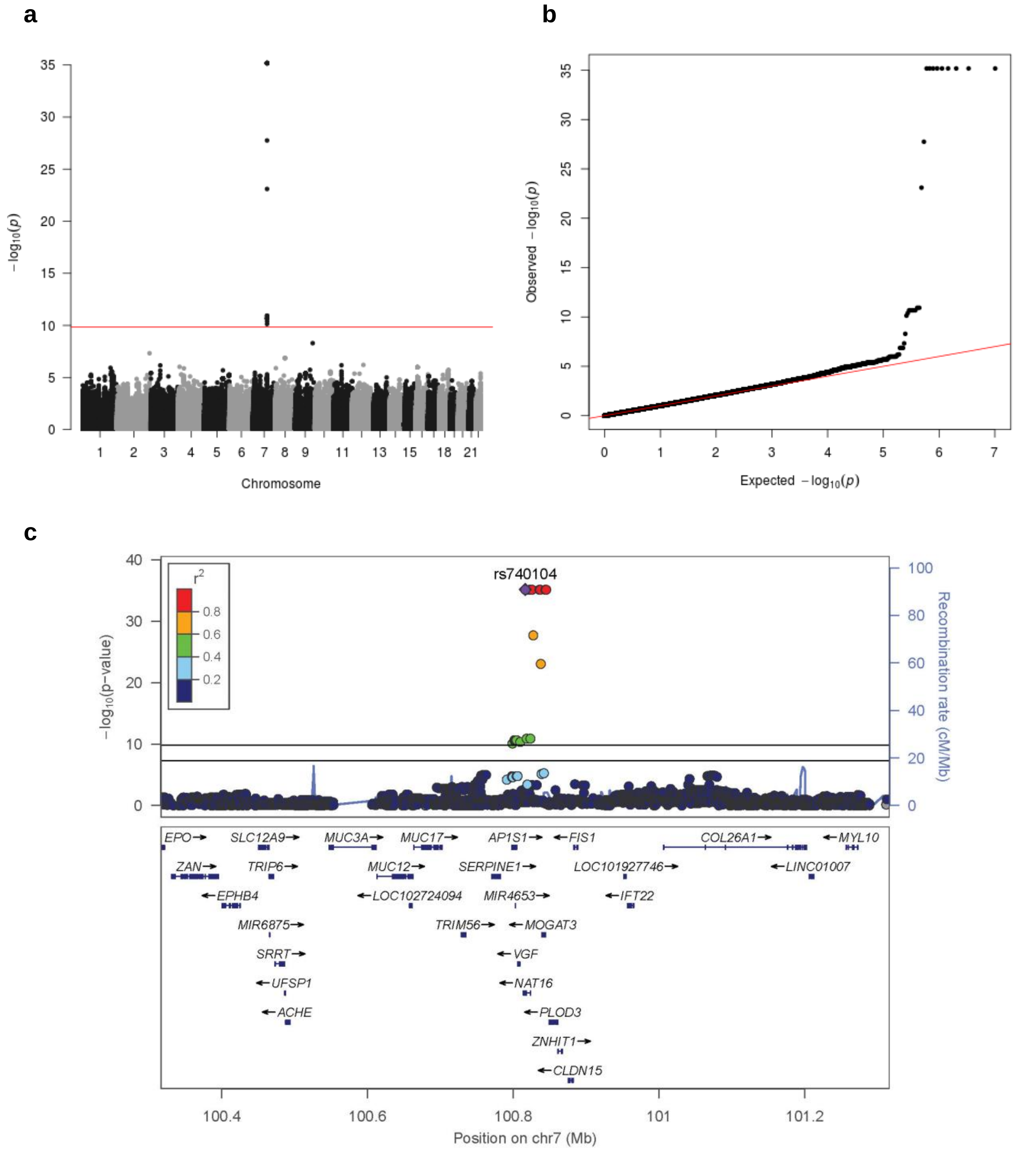

Supplementary Figure 9. GWAS meta-analysis results for N-acetylhistidine (X33946)

a) The Manhattan plot, with a red line indicating the Bonferroni-corrected significance threshold (P = $1.48x10^{-10}$). **b)** The Q-Q plot of the estimated effect sizes. **c)** The regional association plot for the associated locus. The top horizontal line represents the Bonferroni-corrected p-value threshold while the bottom line represents the standard genome-wide significance threshold.

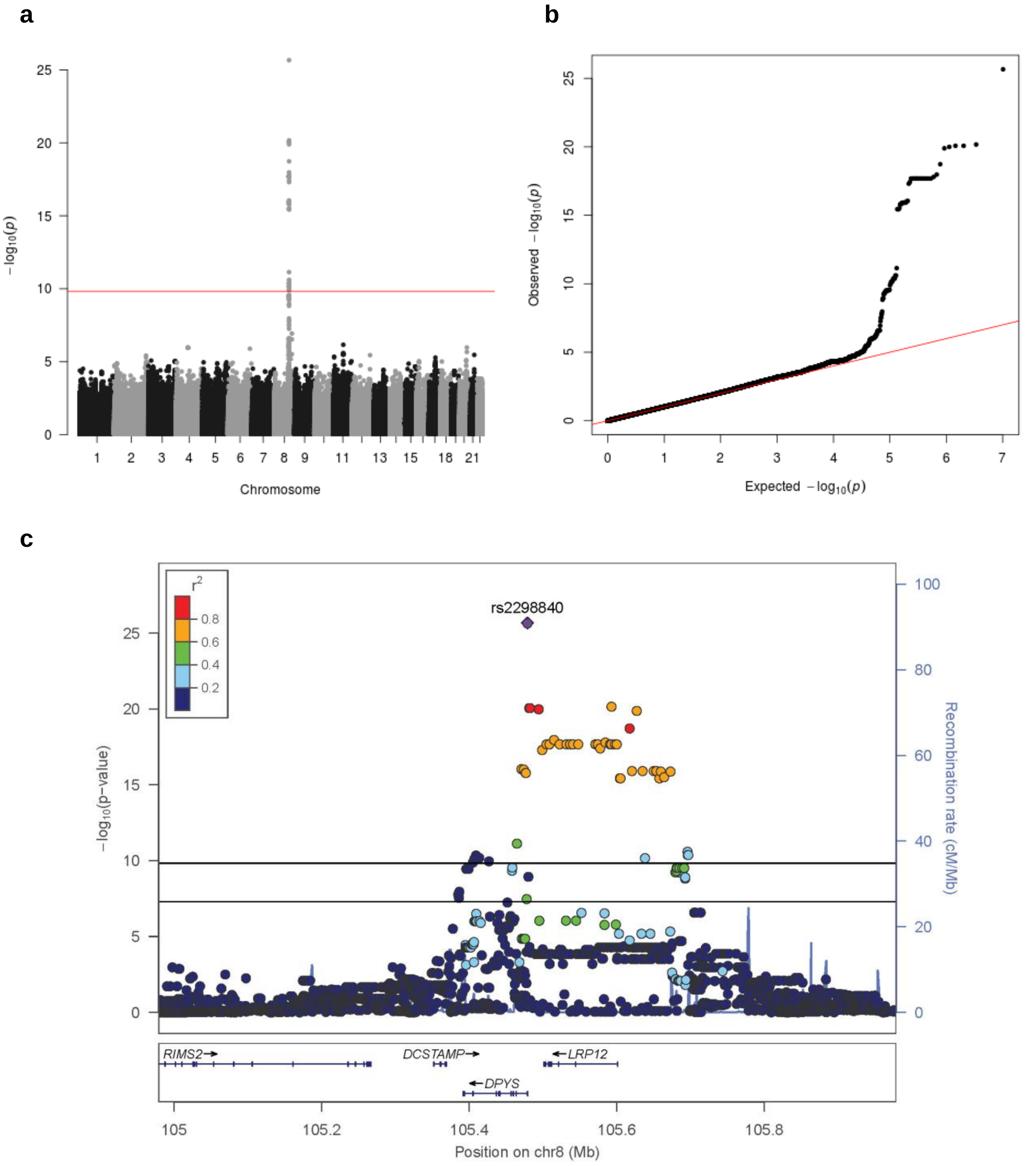

Supplementary Figure 10. GWAS meta-analysis results for 3-ureidopropionate (X3155)

a) The Manhattan plot, with a red line indicating the Bonferroni-corrected significance threshold (P = $1.48\text{x}10^{-10}$). **b)** The Q-Q plot of the estimated effect sizes. **c)** The regional association plot for the associated locus. The top horizontal line represents the Bonferroni-corrected p-value threshold while the bottom line represents the standard genome-wide significance threshold.

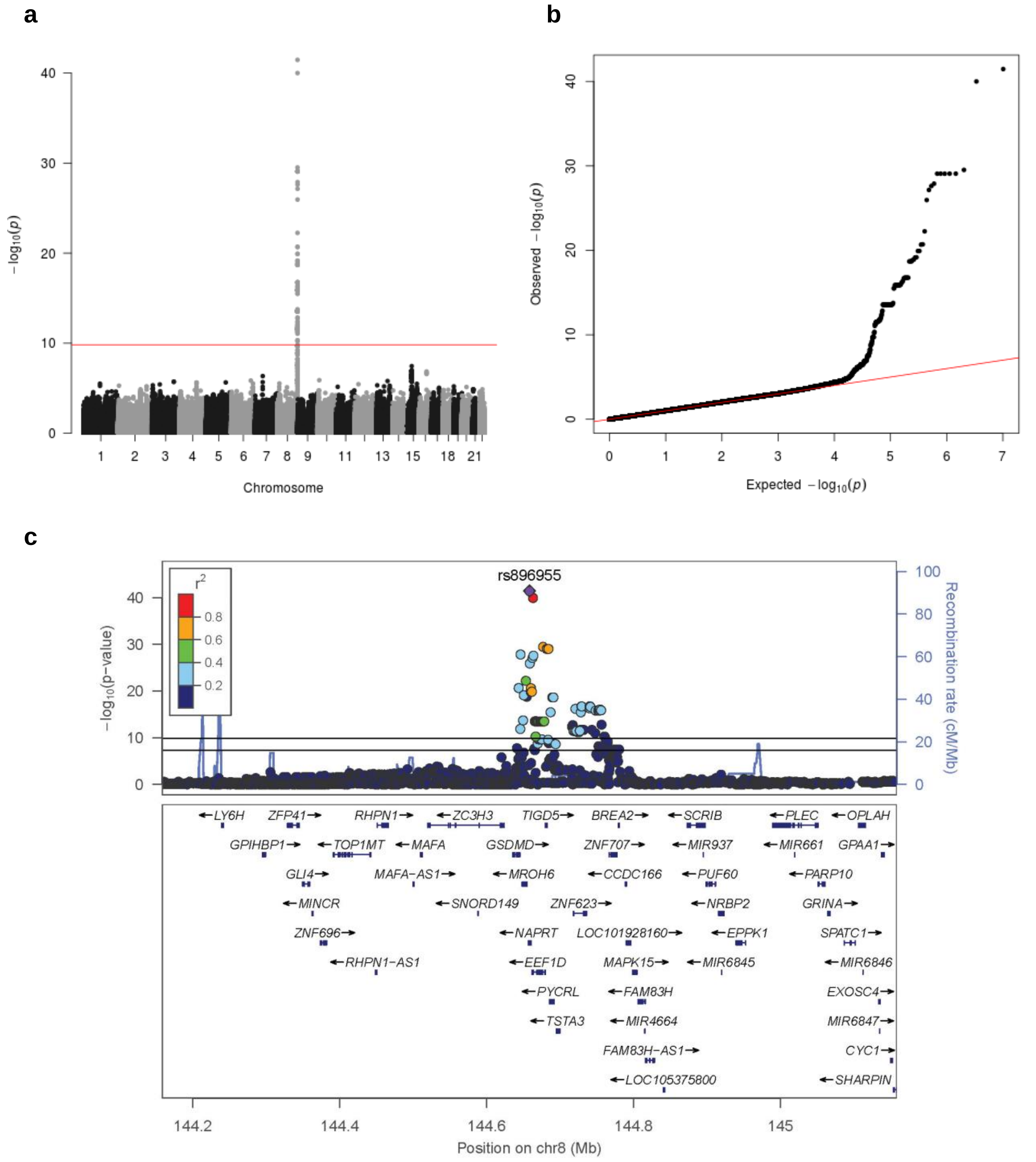

Supplementary Figure 11. GWAS meta-analysis results for 1-ribosyl-imidazoleacetate (X61868)

a) The Manhattan plot, with a red line indicating the Bonferroni-corrected significance threshold (P = 1.48×10^{-10}). **b)** The Q-Q plot of the estimated effect sizes. **c)** The regional association plot for the associated locus. The top horizontal line represents the Bonferroni-corrected p-value threshold while the bottom line represents the standard genome-wide significance threshold.

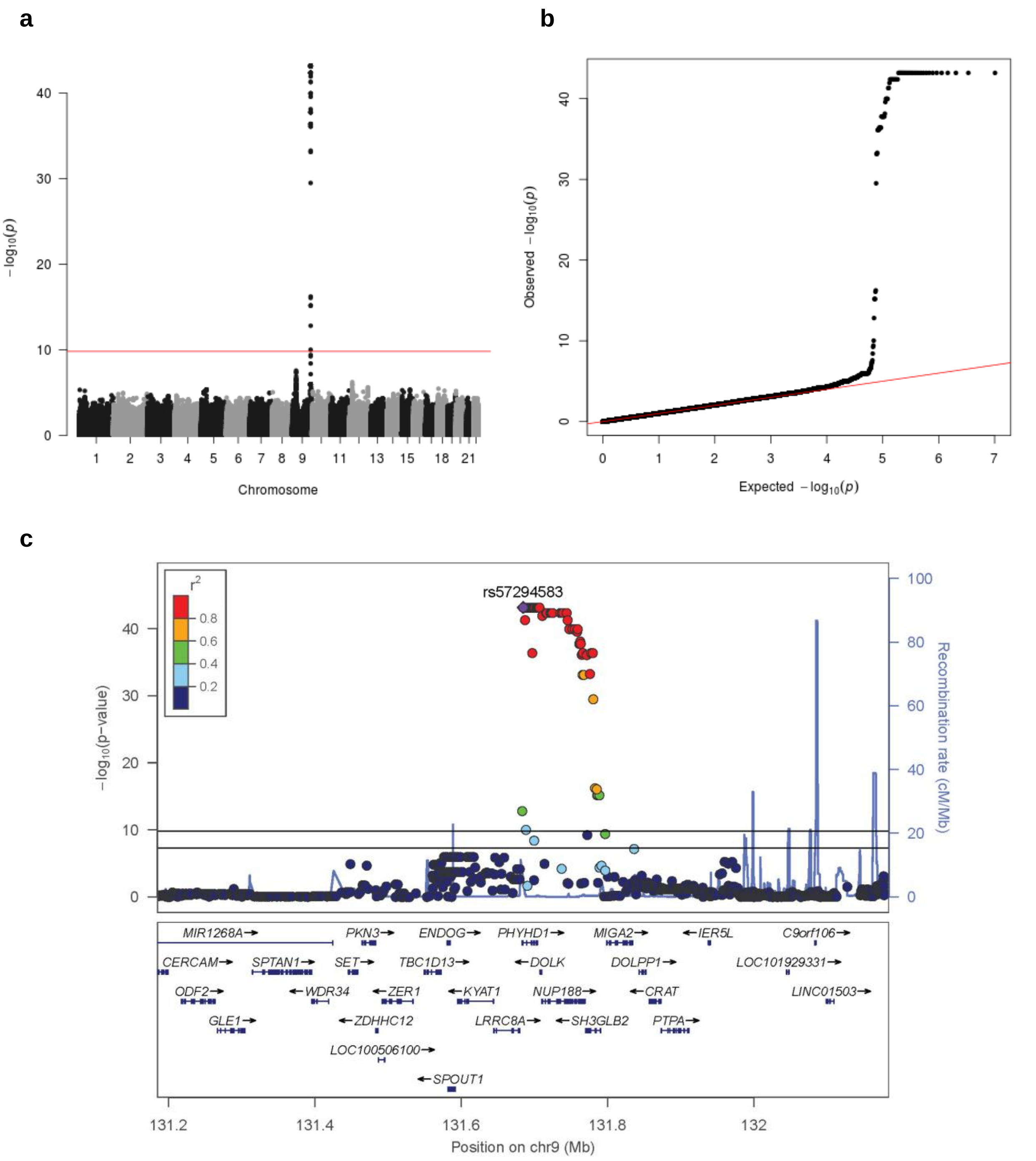

Supplementary Figure 12. GWAS meta-analysis results for 2'-O-methylcytidine (X57554)

a) The Manhattan plot, with a red line indicating the Bonferroni-corrected significance threshold (P = $1.48x10^{-10}$). **b)** The Q-Q plot of the estimated effect sizes. **c)** The regional association plot for the associated locus. The top horizontal line represents the Bonferroni-corrected p-value threshold while the bottom line represents the standard genome-wide significance threshold.

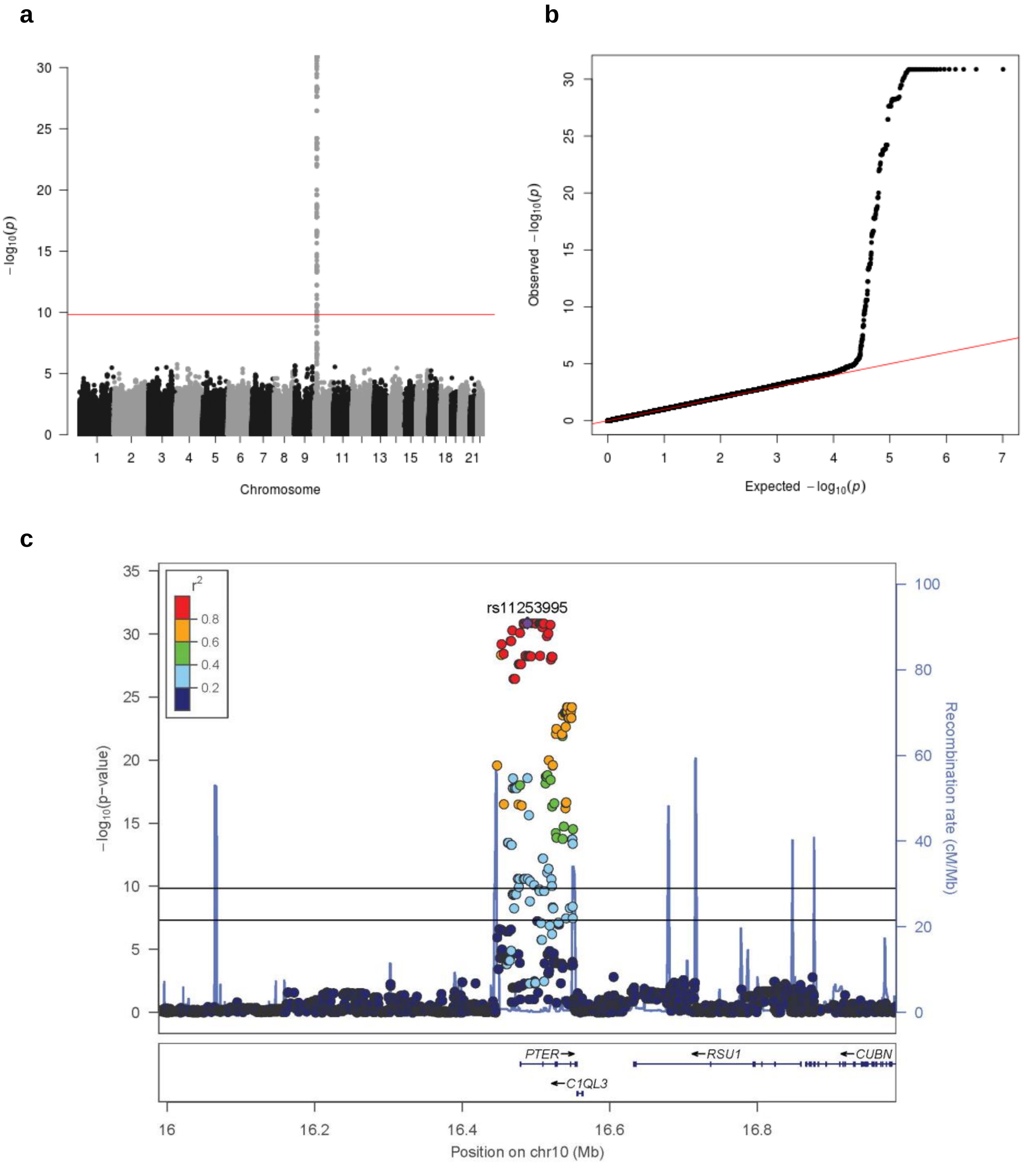

Supplementary Figure 13. GWAS meta-analysis results for N-acetyl-beta-alanine (X37432)

a) The Manhattan plot, with a red line indicating the Bonferroni-corrected significance threshold (P = 1.48×10^{-10}). **b)** The Q-Q plot of the estimated effect sizes. **c)** The regional association plot for the associated locus. The top horizontal line represents the Bonferroni-corrected p-value threshold while the bottom line represents the standard genome-wide significance threshold.

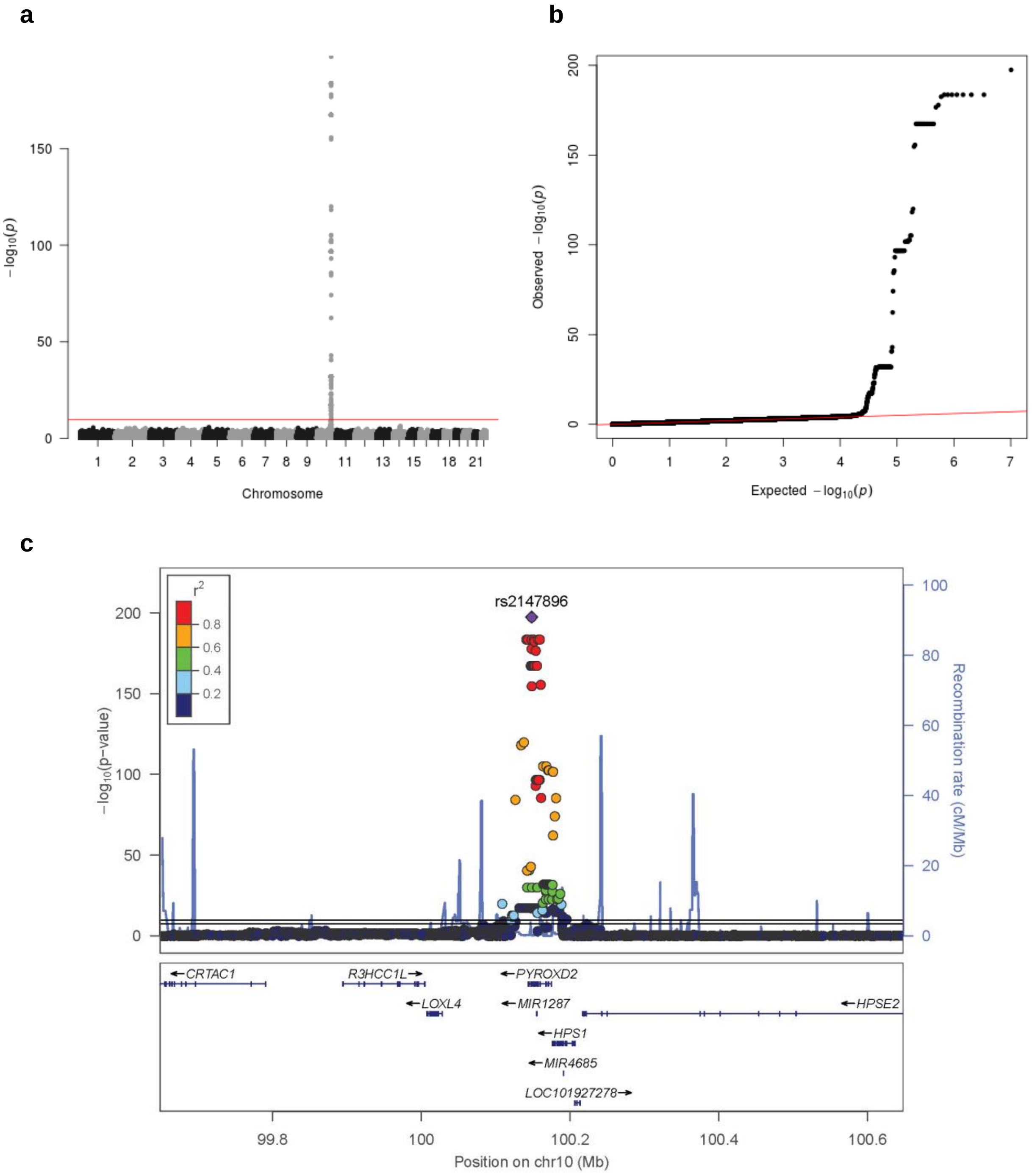

Supplementary Figure 14. GWAS meta-analysis results for N6-methyllysine (X62249)

a) The Manhattan plot, with a red line indicating the Bonferroni-corrected significance threshold (P = $1.48x10^{-10}$). **b)** The Q-Q plot of the estimated effect sizes. **c)** The regional association plot for the associated locus. The top horizontal line represents the Bonferroni-corrected p-value threshold while the bottom line represents the standard genome-wide significance threshold.

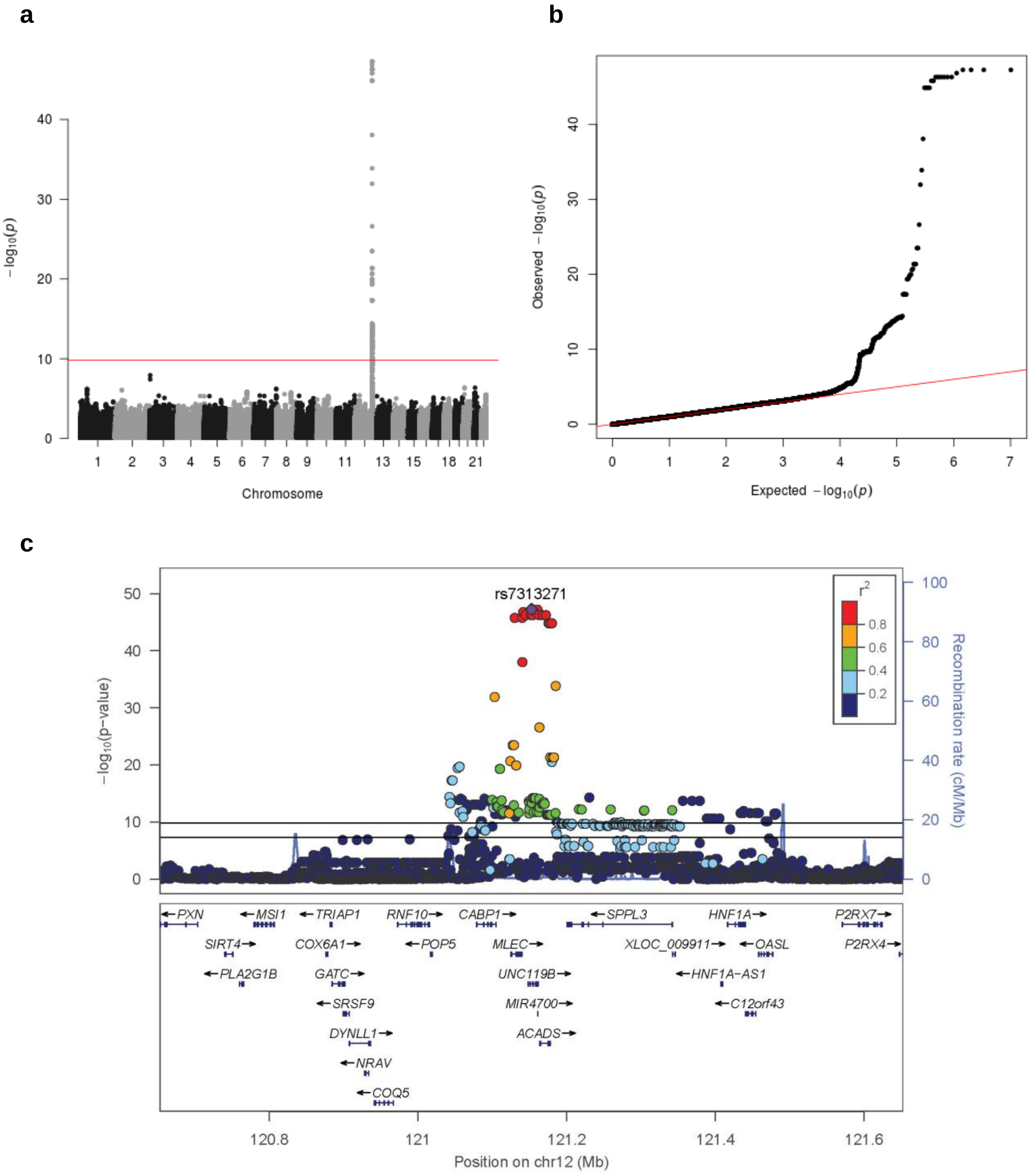

Supplementary Figure 15. GWAS meta-analysis results for ethylmalonate (X15765)

a) The Manhattan plot, with a red line indicating the Bonferroni-corrected significance threshold (P = 1.48×10^{-10}). **b)** The Q-Q plot of the estimated effect sizes. **c)** The regional association plot for the associated locus. The top horizontal line represents the Bonferroni-corrected p-value threshold while the bottom line represents the standard genome-wide significance threshold.

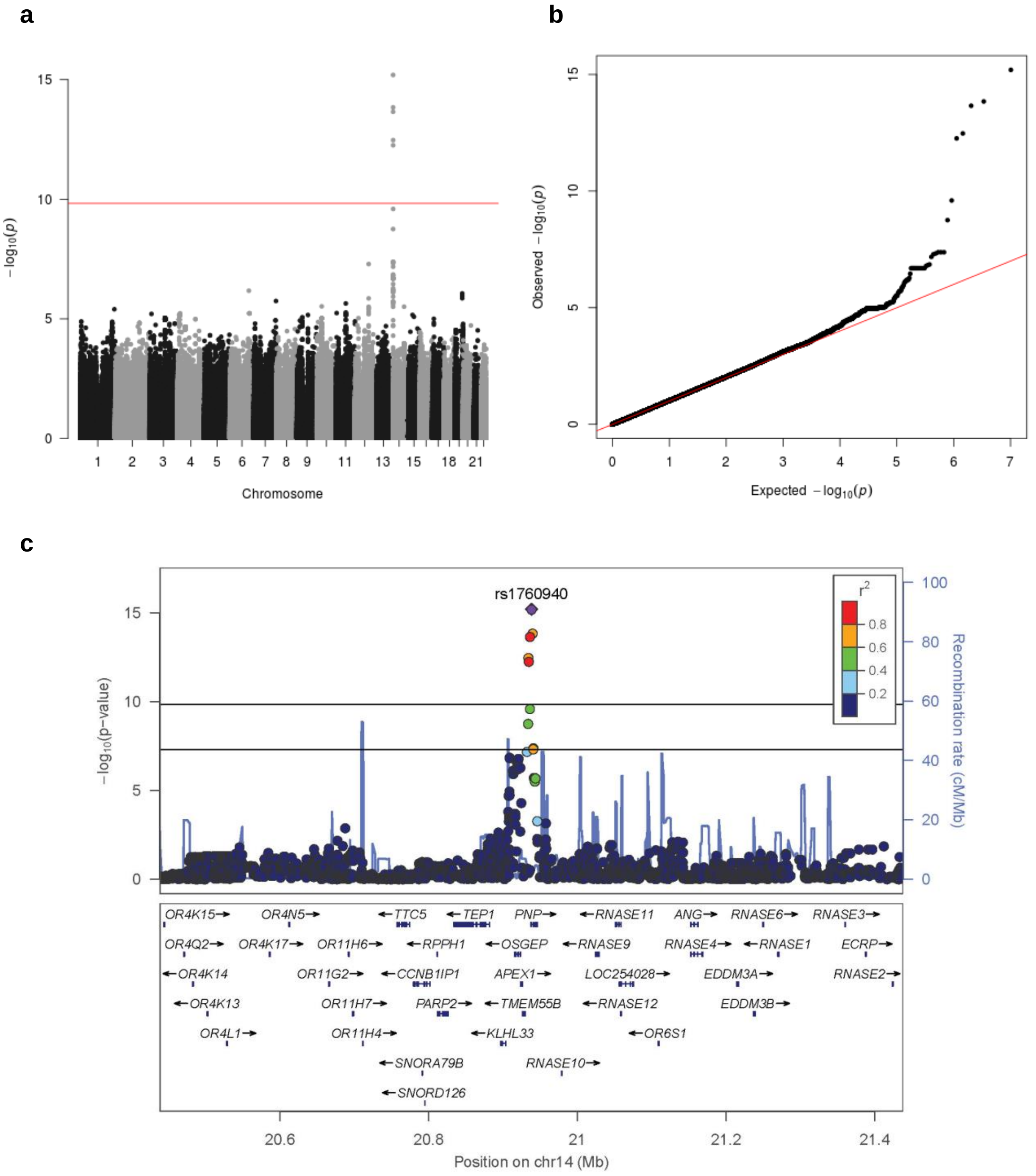

Supplementary Figure 16. GWAS meta-analysis results for guanosine (X1573)

a) The Manhattan plot, with a red line indicating the Bonferroni-corrected significance threshold (P = 1.48×10^{-10}). **b)** The Q-Q plot of the estimated effect sizes. **c)** The regional association plot for the associated locus. The top horizontal line represents the Bonferroni-corrected p-value threshold while the bottom line represents the standard genome-wide significance threshold.

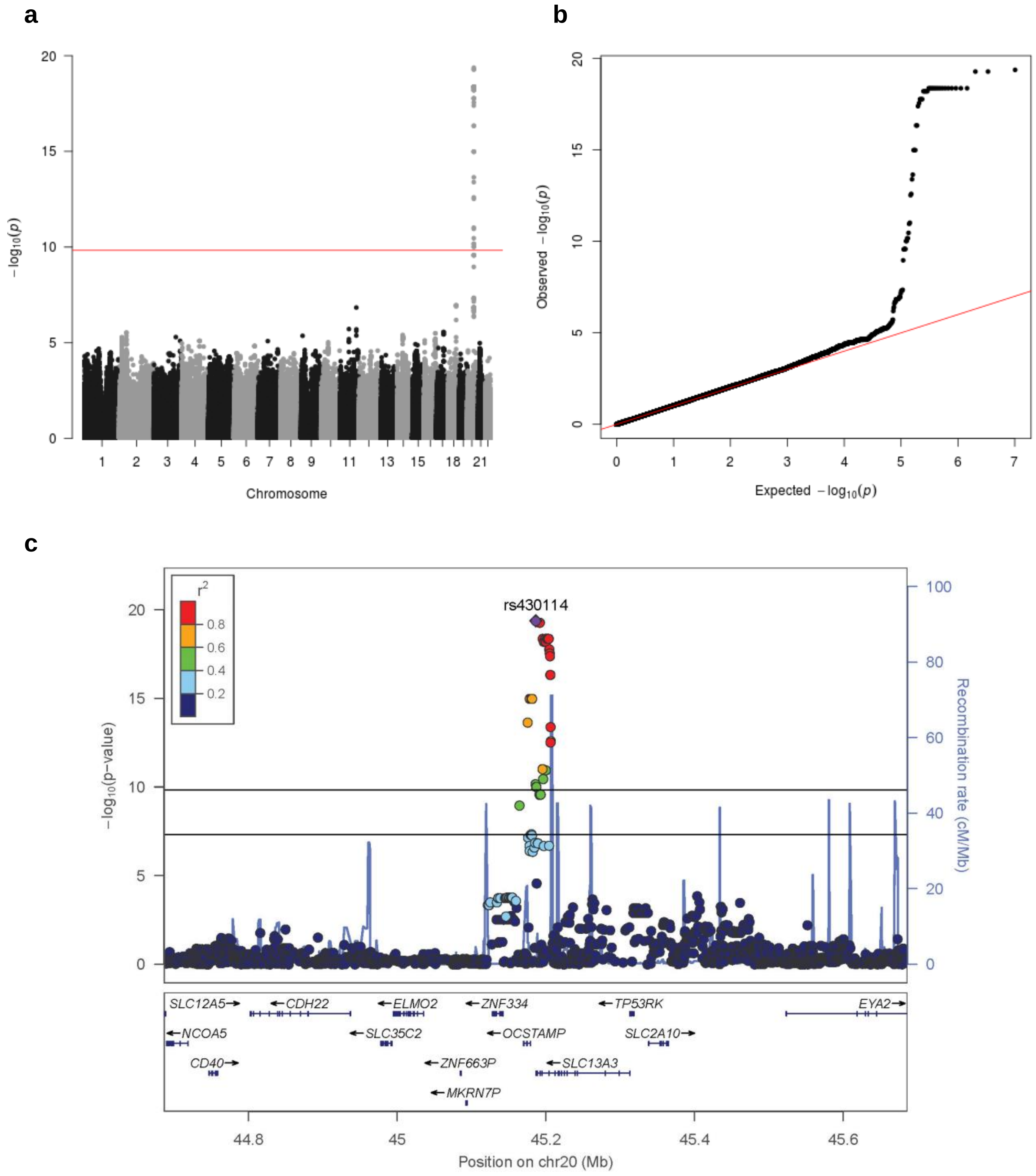

Supplementary Figure 17. GWAS meta-analysis results for N-acetylglutamate (X15720)

a) The Manhattan plot, with a red line indicating the Bonferroni-corrected significance threshold (P = 1.48×10^{-10}). **b)** The Q-Q plot of the estimated effect sizes. **c)** The regional association plot for the associated locus. The top horizontal line represents the Bonferroni-corrected p-value threshold while the bottom line represents the standard genome-wide significance threshold.

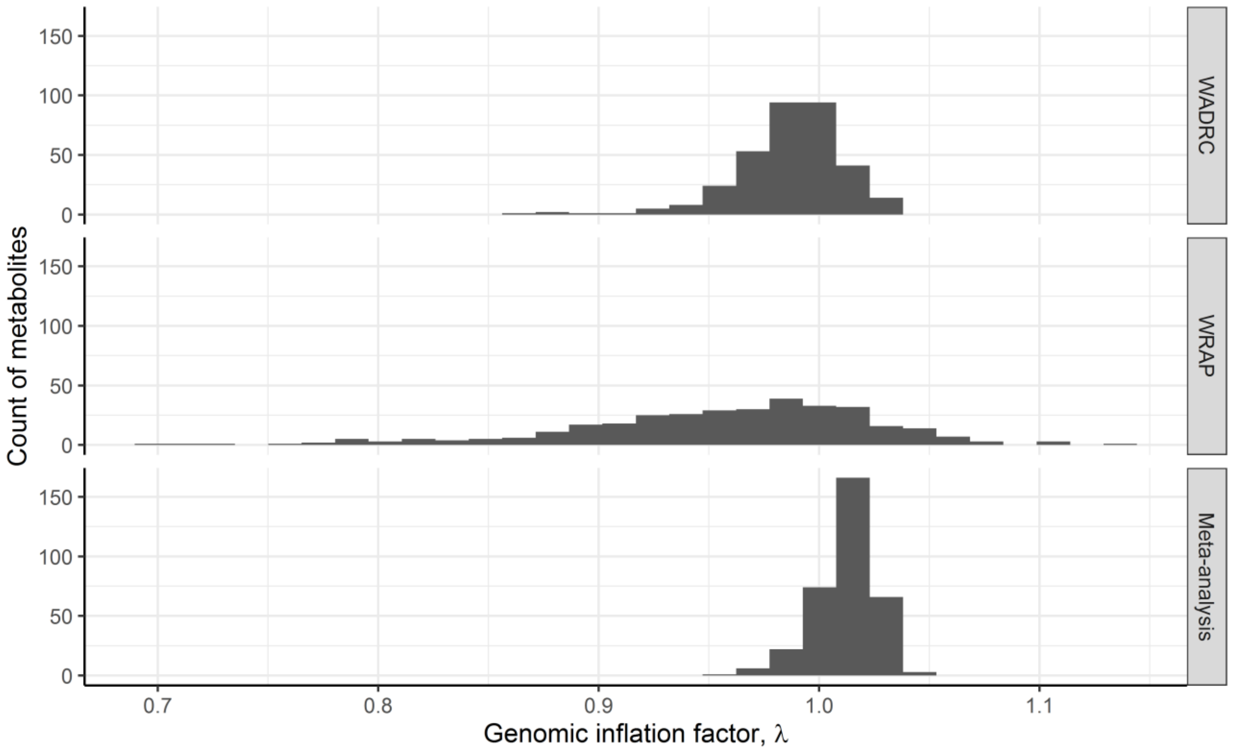

Supplementary Figure 18. Genomic inflation factor distribution

The distribution of the genomic inflation factors from the set of GWAS performed in the discovery (WADRC), replication (WRAP), and meta-analysis analyses is shown above.

a

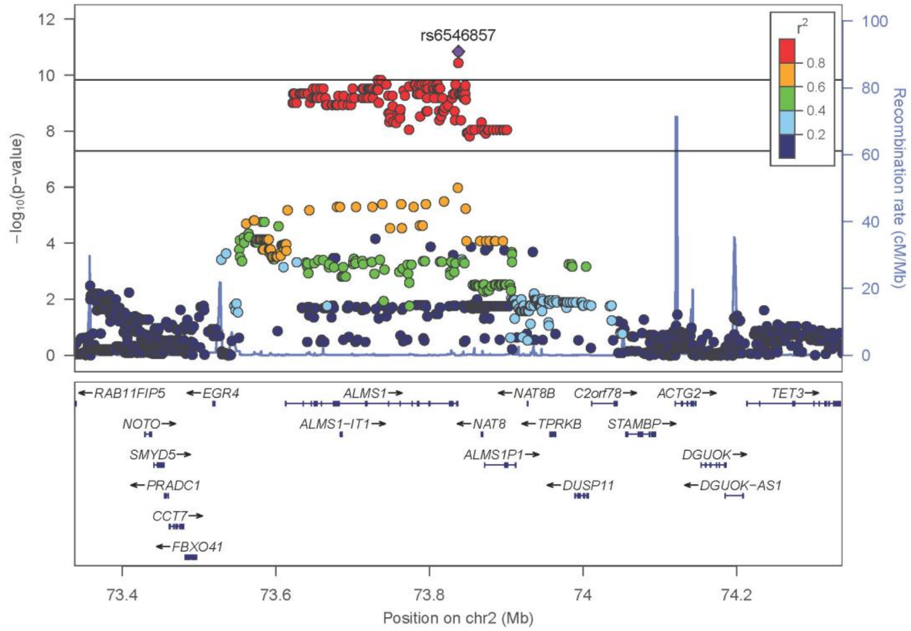

b

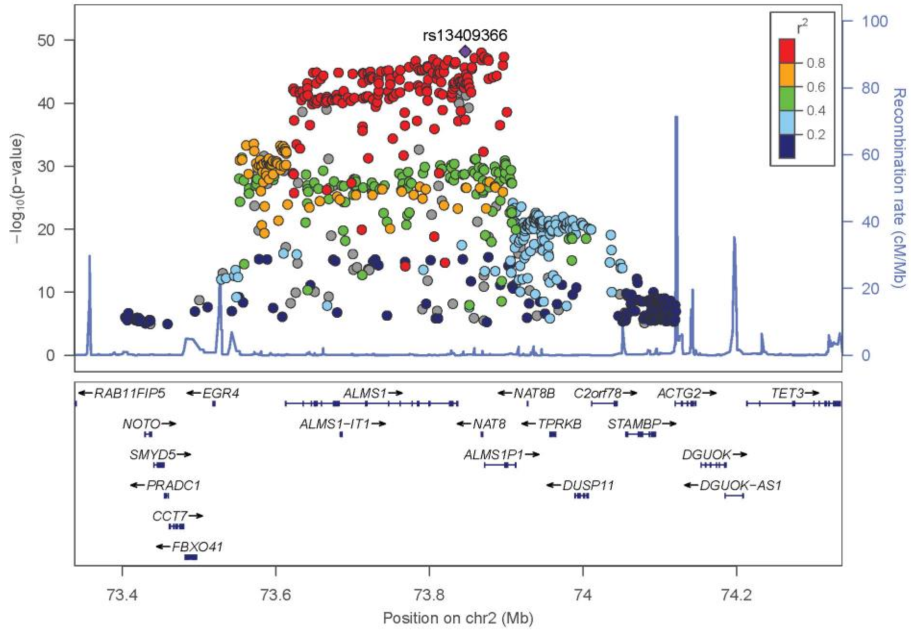

Supplementary Figure 19. Side-by-side regional association plots for methionine sulfone (X44878)

The regional association plots from CSF (**a**) and blood (**b**[1]). In the plot for CSF, the top horizontal line represents the Bonferroni-corrected p-value threshold, while the bottom line represents the standard genome-wide significance threshold. Non-CSF plots were generated manually using publicly available summary statistics.

a

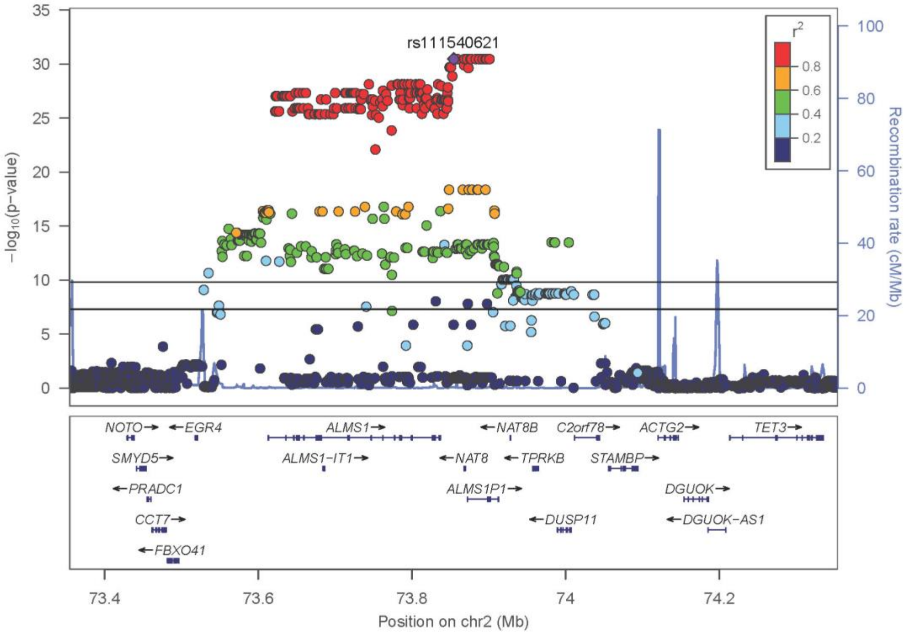

b

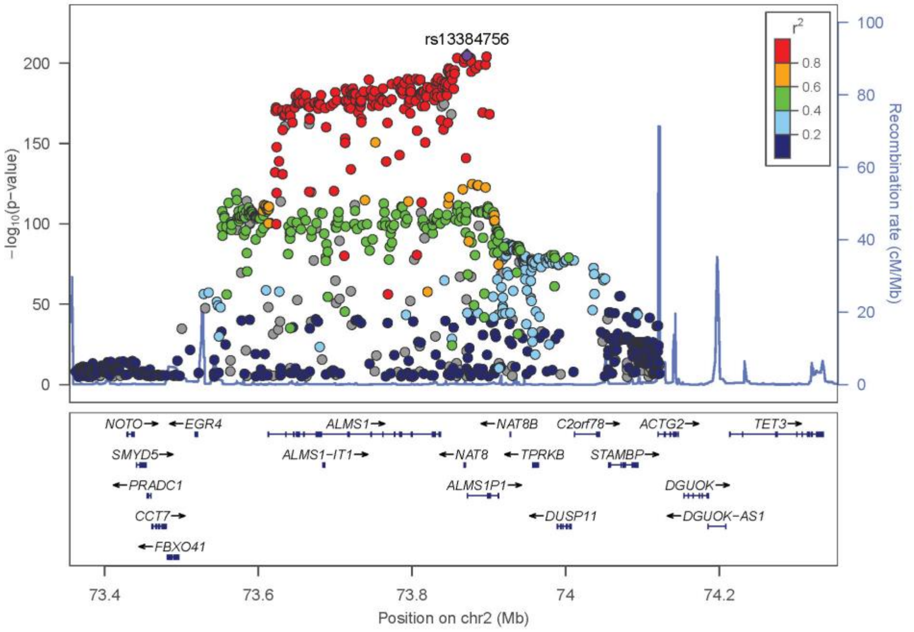

Supplementary Figure 20. Side-by-side regional association plots for N-delta-acetylornithine (X43249)

The regional association plots from CSF (**a**) and blood (**b**[1]). In the plot for CSF, the top horizontal line represents the Bonferroni-corrected p-value threshold, while the bottom line represents the standard genome-wide significance threshold. Non-CSF plots were generated manually using publicly available summary statistics.

a

b

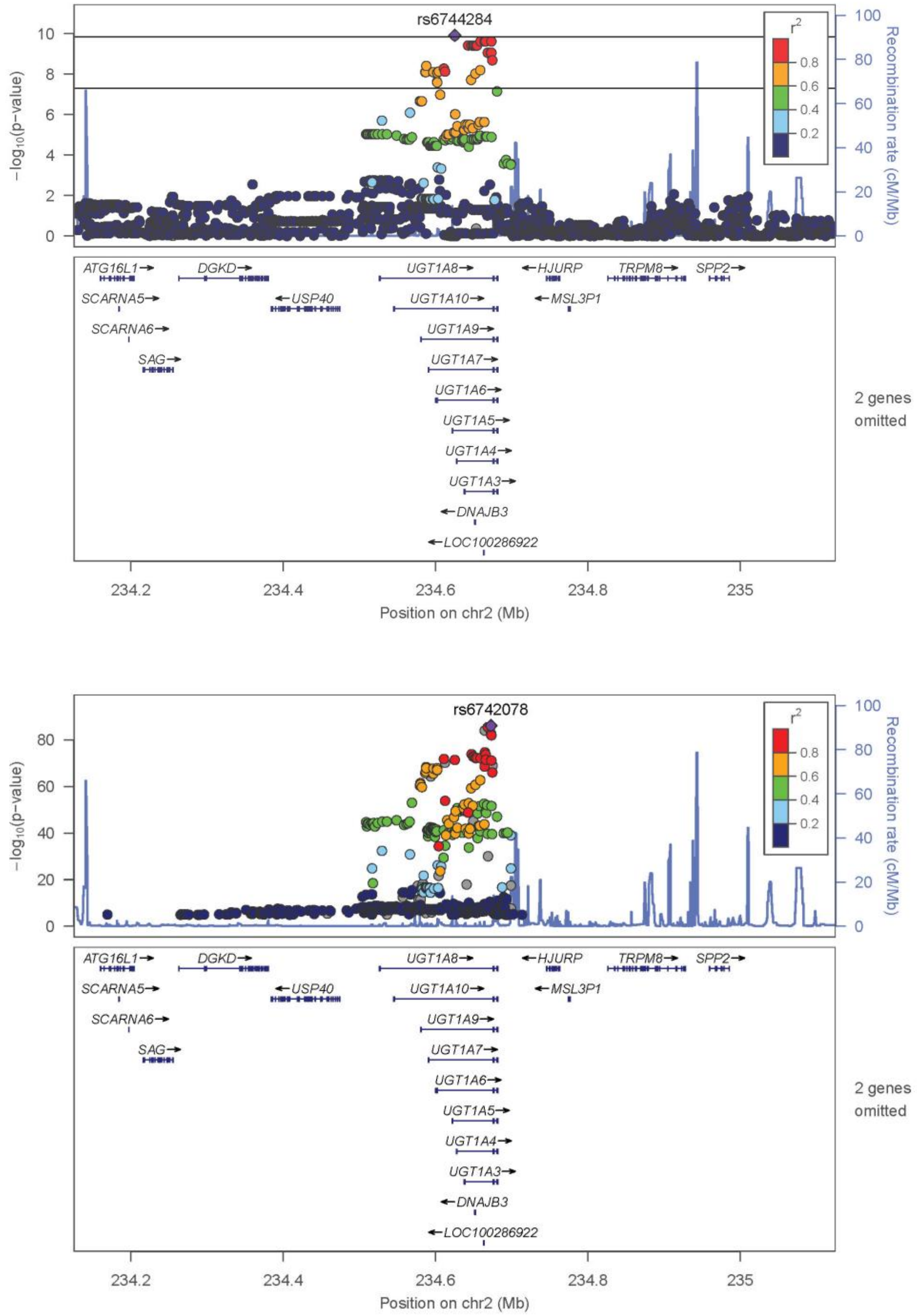

Supplementary Figure 21. Side-by-side regional association plots for bilirubin (X43807)

The regional association plots from CSF (**a**) and blood (**b**[1]). In the plot for CSF, the top horizontal line represents the Bonferroni-corrected p-value threshold, while the bottom line represents the standard genome-wide significance threshold. Non-CSF plots were generated manually using publicly available summary statistics.

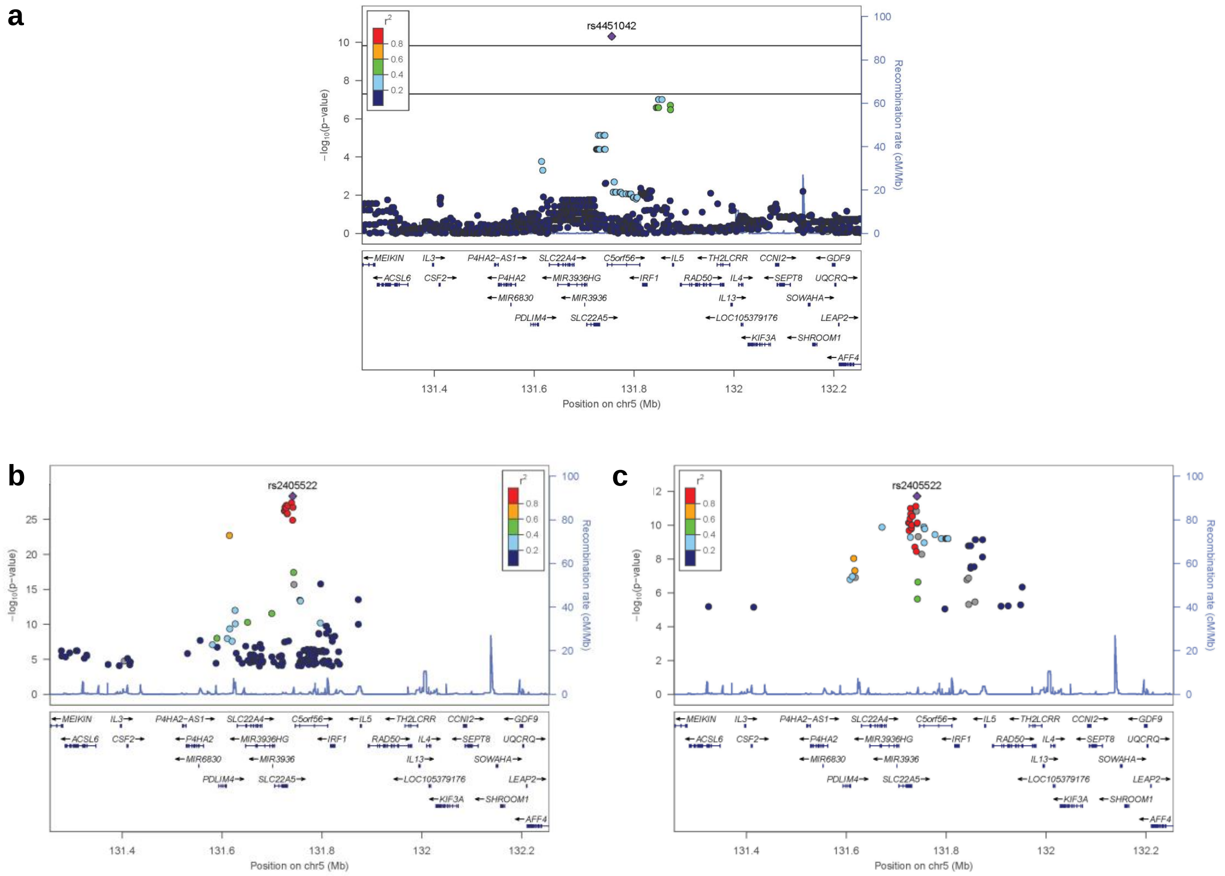

Supplementary Figure 22. Side-by-side regional association plots for tryptophan betaine (X37097)

The regional association plots from CSF (**a**) and blood (**b**[2] and **c**[1]). In the plot for CSF, the top horizontal line represents the Bonferroni-corrected p-value threshold, while the bottom line represents the standard genome-wide significance threshold. Non-CSF plots were generated manually using publicly available summary statistics.

a

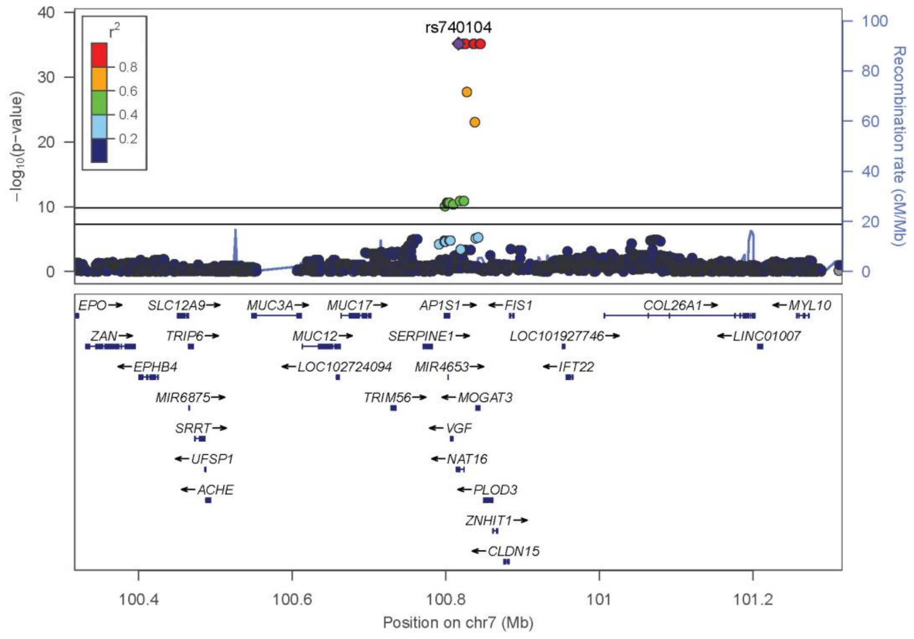

b

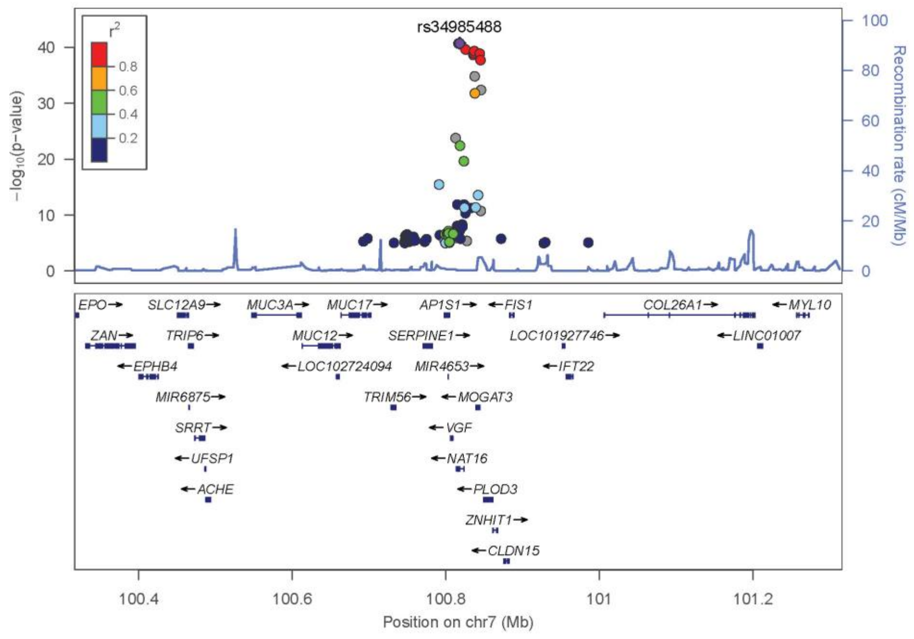

Supplementary Figure 23. Side-by-side regional association plots for N-acetylhistidine (X33946)

The regional association plots from CSF (**a**) and blood (**b**[1]). In the plot for CSF, the top horizontal line represents the Bonferroni-corrected p-value threshold, while the bottom line represents the standard genome-wide significance threshold. Non-CSF plots were generated manually using publicly available summary statistics.

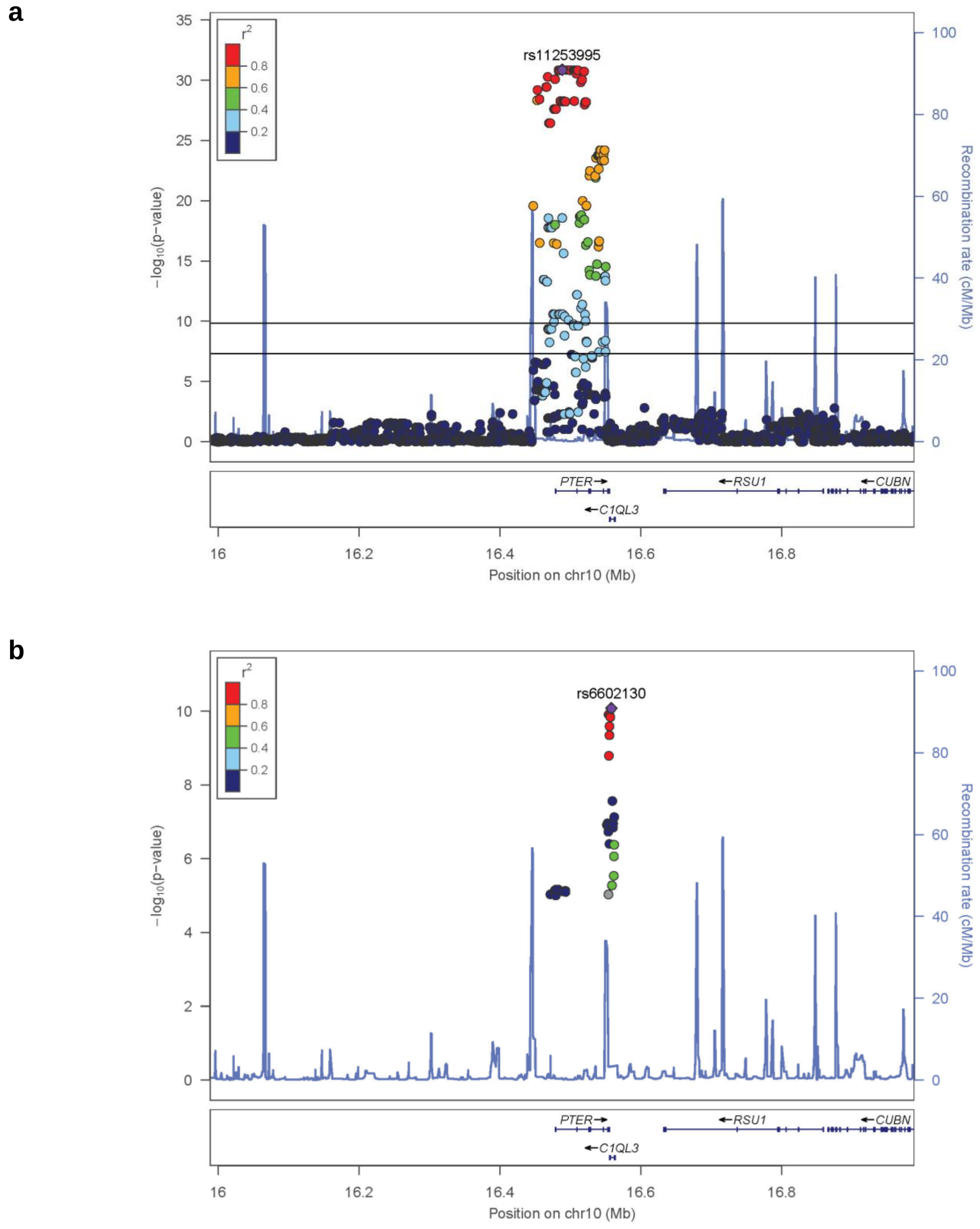

Supplementary Figure 24. Side-by-side regional association plots for N-acetyl-beta-alanine (X37432)

The regional association plots from CSF (**a**) and blood (**b**[1]). In the plot for CSF, the top horizontal line represents the Bonferroni-corrected p-value threshold, while the bottom line represents the standard genome-wide significance threshold. Non-CSF plots were generated manually using publicly available summary statistics.

a

b

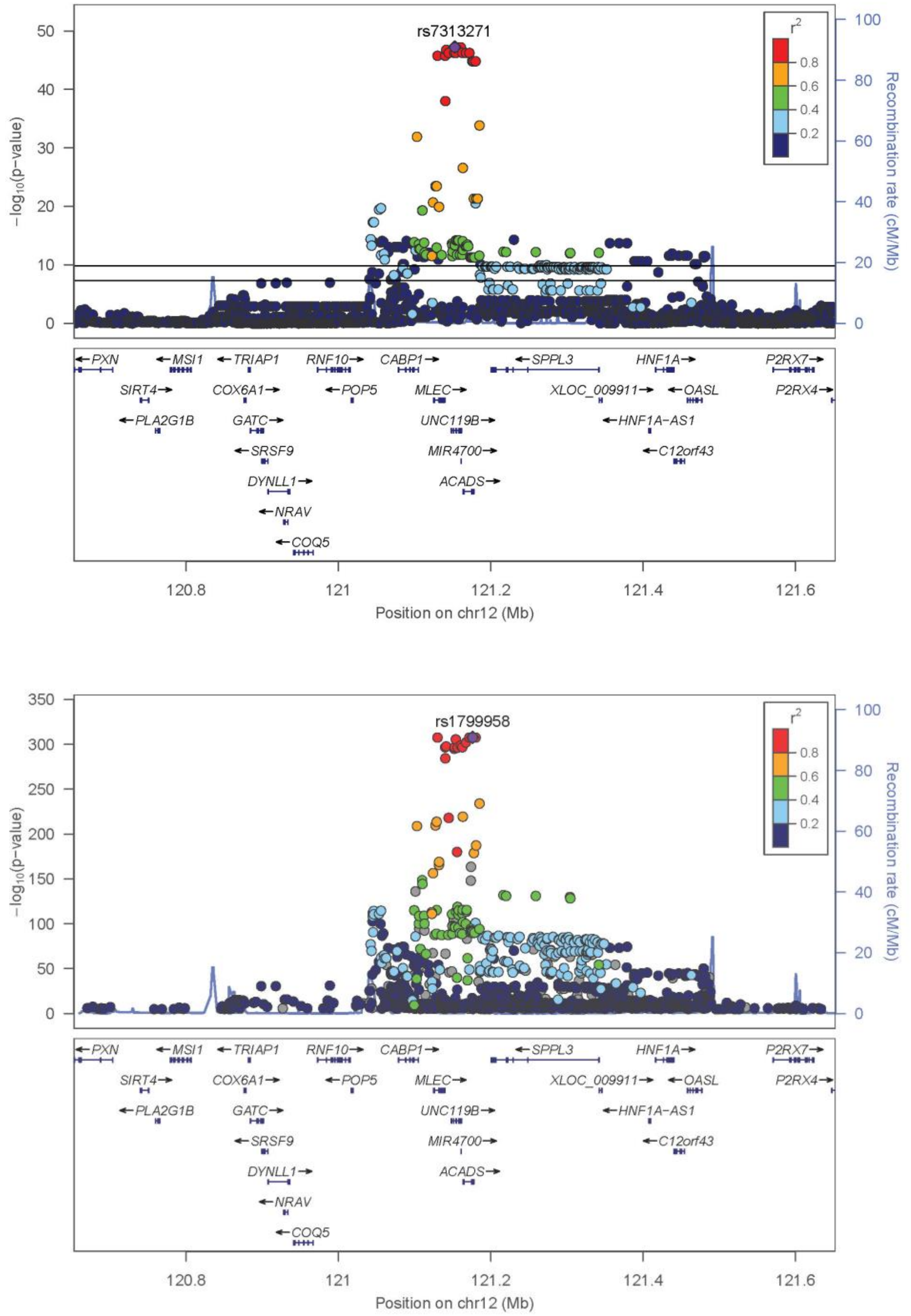

Supplementary Figure 25. Side-by-side regional association plots for ethylmalonate (X15765)

The regional association plots from CSF (**a**) and blood (**b**[1]). In the plot for CSF, the top horizontal line represents the Bonferroni-corrected p-value threshold, while the bottom line represents the standard genome-wide significance threshold. Non-CSF plots were generated manually using publicly available summary statistics.

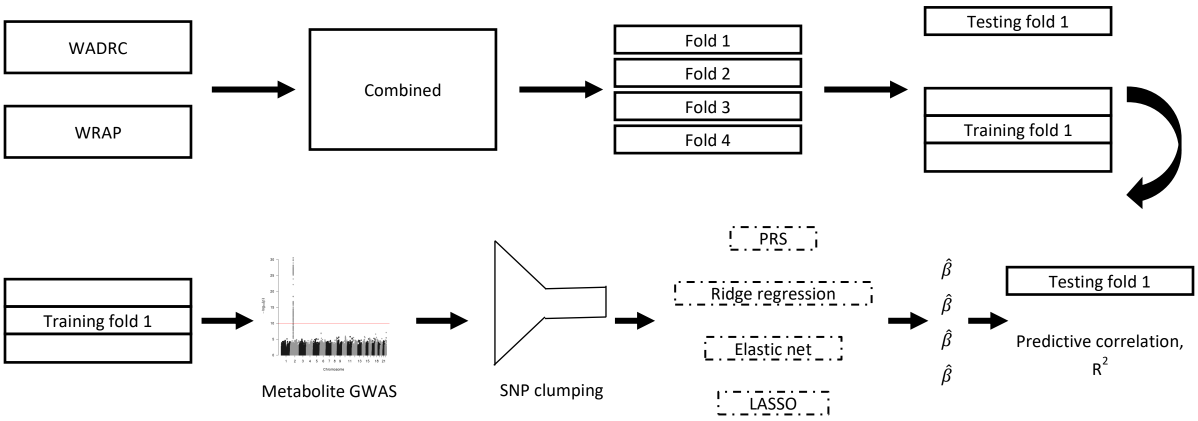

Supplementary Figure 26. Metabolite prediction model building process

The sequence of steps used to build and select each metabolite prediction model are summarized. The combined WADRC and WRAP sample was used to build a variety of predictive models of varying sparsity for each metabolite. Four-fold cross-validation was used to select the best model for each metabolite based on the average predictive correlation across folds.

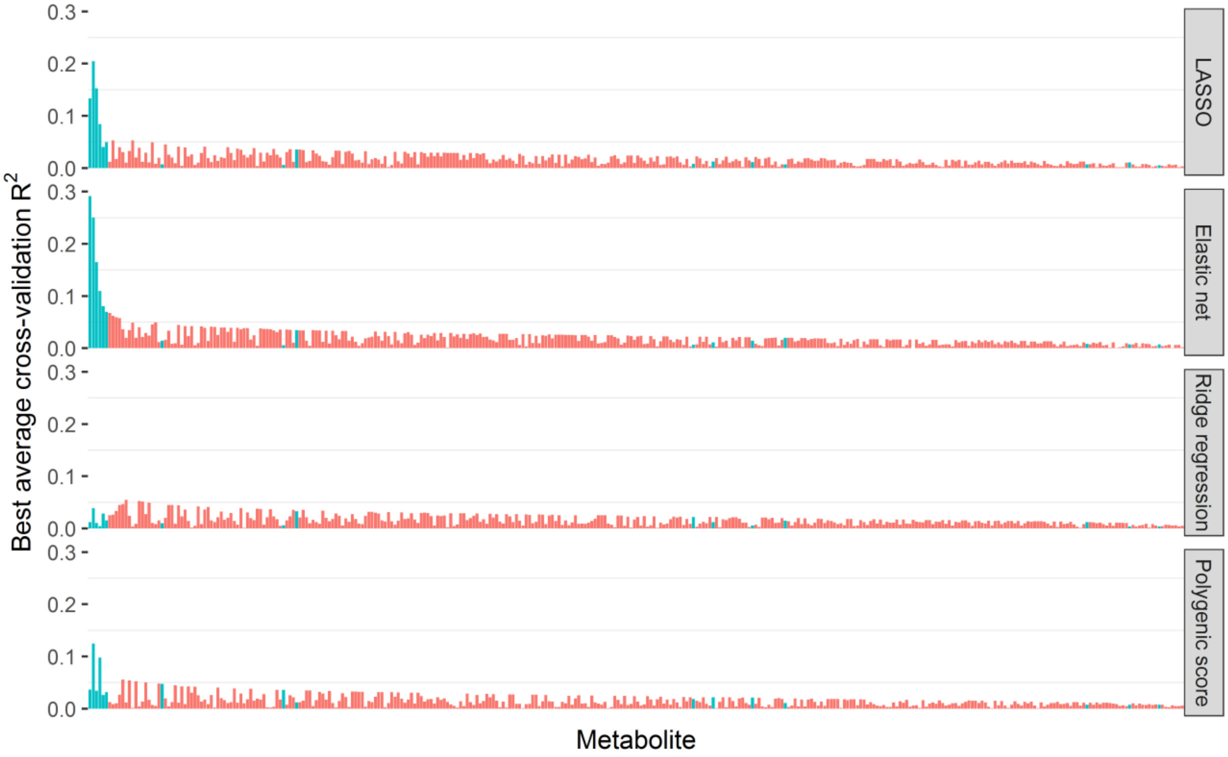

Supplementary Figure 27. Metabolite prediction model performance by model type

The prediction performance of the best model of each model type is shown, with metabolites arranged left-to-right according to the best possible model correlation across all models. Metabolites with a significant locus from the GWAS meta-analysis are highlighted in blue.

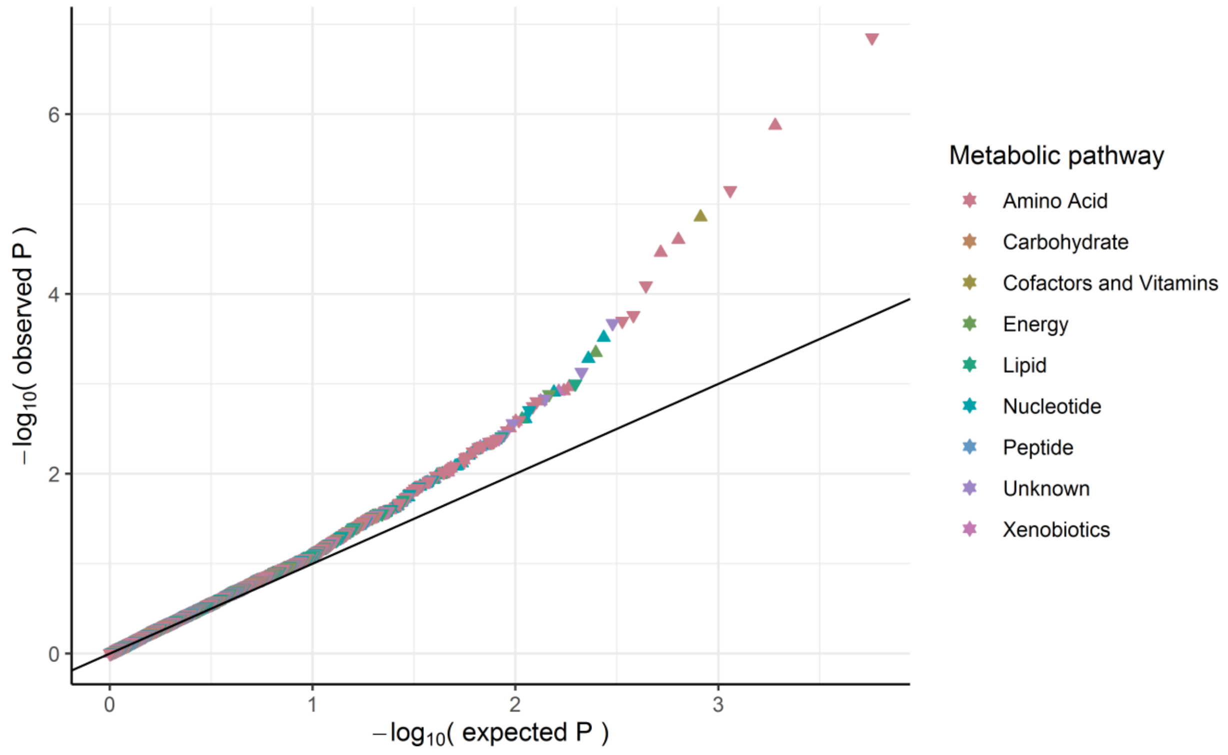

Supplementary Figure 28. Metabolite-phenotype association analysis Q-Q plot

Each point represents the association of one metabolite with a psychiatric or neurological phenotype, with the color representing the metabolite's metabolic pathway. Upward pointing triangles designate a positive association of the metabolite with the phenotype; downward pointing triangles designate a negative association.

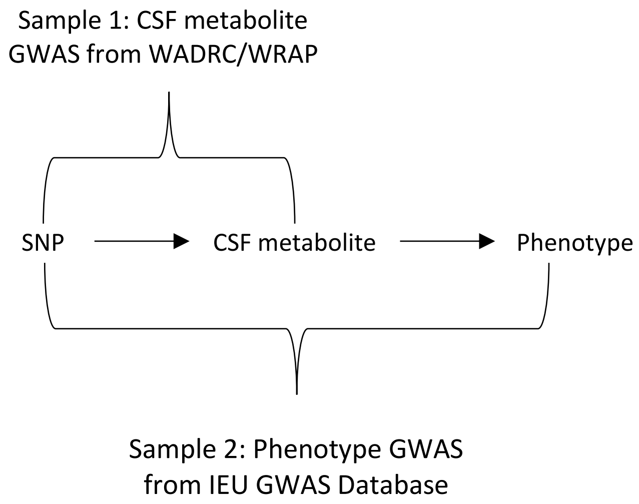

Supplementary Figure 29. Two-sample Mendelian Randomization model set-up

An overview of the sources of each data set used in the two-sample Mendelian Randomization analysis. The set of metabolite-phenotype pairs to analyze was the set of significant metabolite-phenotype associations from the MWAS analysis. For each subsequent MR analysis, the set of instrument SNPs for each metabolite was those that were in the best predictive model for that metabolite from the MWAS analysis; the GWAS summary statistics for the metabolites were those from the WADRC/WRAP GWAS meta-analysis; and the GWAS summary statistics for the phenotypes were taken from summary statistics in the IEU GWAS Database (Supplementary Table 12).

Supplementary References

1. Long, T. *et al.* Whole-genome sequencing identifies common-to-rare variants associated with human blood metabolites. *Nat Genet* **49**, 568–578 (2017).
2. Shin, S.-Y. *et al.* An atlas of genetic influences on human blood metabolites. *Nat Genet* **46**, 543–550 (2014).
