# Supplementary material for: Cerebrospinal fluid metabolomics identifies 19 brain-related phenotype associations
Source: Commun Biol. 2021 Jan 12;4:63. doi: 10.1038/s42003-020-01583-z (PMC7803963; doi:10.1038/s42003-020-01583-z)
Supplement: Supplementary file 3 — Description of Additional Supplementary Files [file 42003_2020_1583_MOESM3_ESM.pdf]

Description of Additional Supplementary Files

File Name: Supplementary Data 1

Description:

Supplementary Table 1: Study cohort description

Supplementary Table 2: Metabolite information

Supplementary Table 3: Top significant SNP-metabolite associations

Supplementary Table 4: GWAS meta-analysis significant SNPs

Supplementary Table 5: GWAS meta-analysis significant eQTLs

Supplementary Table 6: GWAS Catalog results for significant SNPs

Supplementary Table 7: Metabolite predictive model summary

Supplementary Table 8: Metabolite predictive model SNP coefficients

Supplementary Table 9: Neurological and psychiatric GWAS used in BADGERS

Supplementary Table 10: BADGERS association results

Supplementary Table 11: MR analysis results

Supplementary Table 12: Neurological and psychiatric GWAS used in the MR analysis
