# Supplementary material for: Cerebrospinal fluid metabolomics identifies 19 brain-related phenotype associations
Source: Commun Biol. 2021 Jan 12;4:63. doi: 10.1038/s42003-020-01583-z (PMC7803963; doi:10.1038/s42003-020-01583-z)
Supplement: Supplementary file 5 — Reporting Summary [file 42003_2020_1583_MOESM5_ESM.pdf]

nature research

Corresponding author(s): Qiongshi Lu

Last updated by author(s): Nov 21, 2020

Reporting Summary

Nature Research wishes to improve the reproducibility of the work that we publish. This form provides structure for consistency and transparency in reporting. For further information on Nature Research policies, see our Editorial Policies and the Editorial Policy Checklist.

Statistics

For all statistical analyses, confirm that the following items are present in the figure legend, table legend, main text, or Methods section.

n/a	Confirmed	
☐	☒	The exact sample size (n) for each experimental group/condition, given as a discrete number and unit of measurement
☐	☒	A statement on whether measurements were taken from distinct samples or whether the same sample was measured repeatedly
☐	☒	The statistical test(s) used AND whether they are one- or two-sided<br>*Only common tests should be described solely by name; describe more complex techniques in the Methods section.*
☐	☒	A description of all covariates tested
☐	☒	A description of any assumptions or corrections, such as tests of normality and adjustment for multiple comparisons
☐	☒	A full description of the statistical parameters including central tendency (e.g. means) or other basic estimates (e.g. regression coefficient) AND variation (e.g. standard deviation) or associated estimates of uncertainty (e.g. confidence intervals)
☐	☒	For null hypothesis testing, the test statistic (e.g. F, t, r) with confidence intervals, effect sizes, degrees of freedom and P value noted<br>*Give P values as exact values whenever suitable.*
☒	☐	For Bayesian analysis, information on the choice of priors and Markov chain Monte Carlo settings
☒	☐	For hierarchical and complex designs, identification of the appropriate level for tests and full reporting of outcomes
☒	☐	Estimates of effect sizes (e.g. Cohen's d, Pearson's r), indicating how they were calculated

Our web collection on statistics for biologists contains articles on many of the points above.

Software and code

Policy information about availability of computer code

Data collection	This study was a secondary analysis of existing data from the WADRC and WRAP cohorts. No new data collection was performed for this study.
Data analysis	Software packages, version numbers, and relevant citations are provided in the Methods section.

For manuscripts utilizing custom algorithms or software that are central to the research but not yet described in published literature, software must be made available to editors and reviewers. We strongly encourage code deposition in a community repository (e.g. GitHub). See the Nature Research guidelines for submitting code & software for further information.

Data

Policy information about availability of data

All manuscripts must include a data availability statement. This statement should provide the following information, where applicable:

- Accession codes, unique identifiers, or web links for publicly available datasets
- A list of figures that have associated raw data
- A description of any restrictions on data availability

The datasets generated and analyzed during the current study may be requested from the WADRC at https://www.adrc.wisc.edu/apply-resources. Full GWAS meta-analysis summary statistics may be accessed at ftp://ftp.biostat.wisc.edu/pub/lu_group/Projects/MWAS/.

April 2020

Field-specific reporting

Please select the one below that is the best fit for your research. If you are not sure, read the appropriate sections before making your selection.

☒ Life sciences ☐ Behavioural & social sciences ☐ Ecological, evolutionary & environmental sciences

For a reference copy of the document with all sections, see nature.com/documents/nr-reporting-summary-flat.pdf

Life sciences study design

All studies must disclose on these points even when the disclosure is negative.

Sample size	This study was a secondary data analysis of existing data from the WADRC and WRAP cohorts. The sample sizes analyzed here were determined by the number of samples with the necessary data from the cohorts and meeting the criteria for this analysis, as described in the Methods section.
Data exclusions	As described in the Methods section, the analysis was restricted to unrelated individuals who were cognitively healthy at baseline. The reason for the relatedness exclusion was to allow for simple linear models to suffice in the GWAS. The reason for the exclusion of non-cognitively healthy samples was to improve the generalizability of the metabolite prediction models. Individuals of non-European ancestry were excluded due to insufficient statistical power for the analyses in this study.
Replication	A discovery/replication analysis was used for the GWAS of the CSF metabolites to ensure consistent effects across the WADRC and WRAP cohorts.
Randomization	The major covariates (age at sample, sex, principal components of ancestry, and genotyping batch) were controlled for in the GWAS analyses.
Blinding	There was no experimental group allocation that was relevant to this study.

Reporting for specific materials, systems and methods

We require information from authors about some types of materials, experimental systems and methods used in many studies. Here, indicate whether each material, system or method listed is relevant to your study. If you are not sure if a list item applies to your research, read the appropriate section before selecting a response.

Materials & experimental systems

n/a	Involved in the study	
☒	☐	Antibodies
☒	☐	Eukaryotic cell lines
☒	☐	Palaeontology and archaeology
☒	☐	Animals and other organisms
☐	☒	Human research participants
☒	☐	Clinical data
☒	☐	Dual use research of concern

Methods

n/a	Involved in the study	
☒	☐	ChIP-seq
☒	☐	Flow cytometry
☒	☐	MRI-based neuroimaging

Human research participants

Policy information about studies involving human research participants

Population characteristics	This study is a secondary data analysis of data from the WADRC and WRAP studies, described below:<br><br>The metabolomics data used in this study came from CSF samples analyzed in the WADRC and WRAP cohort studies. The WADRC, previously described, is a longitudinal cohort study of memory, aging, and AD in middle and older aged adults who were recruited into one of six subgroups: 1) mild late-onset AD; 2) mild cognitive impairment (MCI); 3) age-matched healthy older controls (age > 65); 4) middle-aged adults with a positive parental history of AD; 5) middle-aged adults with a negative parental history of AD; and 6) middle-aged adults with indeterminate parental history of AD. The National Institute of Neurological and Communicative Disorders and Stroke and Alzheimer's Disease and Related Disorders Association (NINCDS-ADRDA) and National Institute on Aging and Alzheimer's Association (NIA-AA) criteria were used for clinical diagnoses. Briefly, the inclusion criteria for WADRC participants included an age ≥ 45, decisional capacity, and the ability to fast from food and drink for 12 hours. Briefly, exclusion criteria included history of certain medical conditions (e.g., kidney dysfunction, congestive heart failure, major neurologic disorders other than dementia, and others), lack of a study partner, and contraindication to biomarker procedures.<br><br>The WRAP study, also previously described, is a longitudinal cohort study of AD in middle and older aged adults who are cognitively healthy at baseline, enriched for persons with a parental history of AD. Generally, inclusion criteria include being between the ages of 40 and 65, fluent in English, able to complete neuropsychological testing, and free of health conditions

	that might preclude study participation. Generally, exclusion criteria included having a diagnosis or evidence of dementia at baseline.
Recruitment	See the above description of the population characteristics for details on the recruitment. Because the WADRC and WRAP cohorts focus on Alzheimer's disease, there could be a bias toward individuals at risk for Alzheimer's disease. However, for this reason, we restricted the analyses to individuals who were cognitively healthy in order to reduce the potential for bias from studying a population that would have included many individuals with cognitive symptoms. Other potential sources of bias could be self-selection into the studies.
Ethics oversight	This study was performed as part of the Generations of WRAP (GROW) study, which was approved by the University of Wisconsin Health Sciences Institutional Review Board. Participants in the WADRC and WRAP studies provided written informed consent.

Note that full information on the approval of the study protocol must also be provided in the manuscript.
